# Experimental and DFT/TD-DFT Insights into Spectroscopic, Solvatochromic, and Nonlinear Optical Properties of Methoxy-Substituted *N*‑Benzylideneaniline Schiff Base Regioisomers

**DOI:** 10.1021/acsomega.6c02717

**Published:** 2026-07-08

**Authors:** Wilson Bosco Paul Michael, Hemamalini Abraham, Selvam Amudhan Senthan, Carlson Alexander, A. Irudaya Jothi

**Affiliations:** † Department of Chemistry, Sacred Heart College, Tirupattur, Tamil Nadu 635601, India; ‡ Department of Chemistry, 30011 St. Joseph’s College, Tiruchirappalli (Affiliated to Bharathidasan University, Tiruchirappalli 620024), Tiruchirappalli, Tamil Nadu 620002, India; § 29843 NAWaL Analytical Laboratories India Pvt. Ltd., Electrical & Electronics Industrial Estate, Hosur, Tamil Nadu 635109, India; ∥ Department of Chemistry, 26679 Hong Kong Baptist University, Ho Sing Hang Campus, 224 Waterloo Road, Kowloon Tong, Hong Kong SAR 999077, China

## Abstract

Schiff bases bearing donor–acceptor substituents represent versatile D–*π*–A systems with tunable electronic and optical properties. Herein, we report combined experimental spectroscopic and DFT/TD-DFT (B3LYP/6-311++G­(d,p)) investigations of methoxy-substituted *N*-benzylideneaniline Schiff base regioisomers, benchmarked against unsubstituted analogues, revealing substituent position–controlled spectral and nonlinear optical properties. The FT-IR and NMR results demonstrate that substituent identity and position significantly influence the vibrational frequencies of characteristic IR bands and shielding/deshielding patterns of proton and carbon nuclei. UV–Vis and TD-DFT analyses reveal bathochromic shifts of the *π* → π* transitions in polar media with solvatochromic sensitivity decreasing in the order: *ortho* > *para* ≫ *meta*. Molecular electrostatic potential and Mulliken charge analyses indicate that methoxy substitution on the benzylidene ring enhances resonance donation and conjugation in the *ortho*- and *para*-isomers, leading to higher molecular electron polarization and consequently stronger bathochromic shifts and solvatochromic responses than the *meta*-isomer and unsubstituted analogues. Frontier molecular orbital (FMO) analysis further confirms that substituent position and donor strength critically govern the optical response and chemical reactivity of these systems. DFT-calculated polarizabilities reveal that donor–acceptor strength tuned by π-conjugation significantly enhances the NLO responses of these systems. The positional methoxy substitution provides an effective strategy for tuning the electronic and photophysical properties of Schiff bases, highlighting their potential for nonlinear optical and photonic applications.

## Graphical abstract

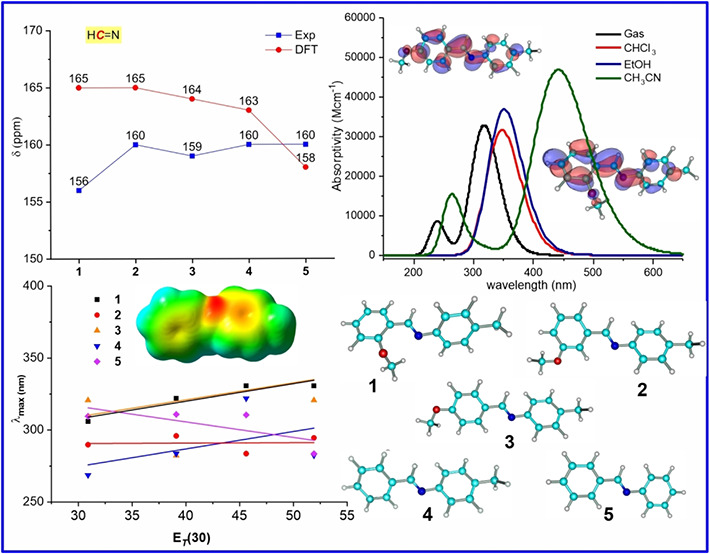

## 1 Introduction

Schiff bases, first reported by Hugo Schiff in 1864,^1^ are among the most versatile and extensively studied organic compounds in making artistic molecules^2^ and in the design of functional molecules with broad relevance to coordination chemistry.^3–5^ Owing to their tunable electronic and structural attributes, Schiff bases and their metal complexes have been extensively investigated as fluorescent chemosensors for metal-ion detection and optical sensing,^6–11^ heavy-metal ion remediation,^12–15^ and in optoelectronic^16,17^ and biomedical applications.^18,19^ *N*-Benzylideneaniline Schiff bases possessing a conjugated aromatic–imine–aromatic (Ar′–CH=N–Ar) framework exhibit optical properties that are highly sensitive to their electronic structure.^20–22^ Subtle structural variations and solvent effects can significantly modulate their spectral characteristics.^23–25^ This sensitivity is further manifested in their pronounced solvatochromic behavior, driven by the interplay of electron-donating (−OH, −OCH_3_, −NH_2_, and alkyl groups) and electron-withdrawing (−NO_2_, Cl, and CN) substituents on the aromatic rings.^26–30^ The resulting molecular asymmetry perturbs π-electron distribution within the conjugated framework, giving rise to bathochromic or hypsochromic shifts depending on solvent polarity and hydrogen-bonding capability.^31–35^ In polar media, enhanced charge redistribution across the imine chromophore leads to pronounced solvatochromic shifts governed by both nonspecific polarity effects and specific solute–solvent interactions.^36–39^ These effects provide mechanistic insight into excited-state charge redistribution and underscore Schiff bases as promising tunable chromophores for photophysical and sensing applications.^31–33^

Systematic studies addressing how positional methoxy substitution modulates molecular electronic dynamics in *N*-benzylideneaniline Schiff bases and their spectroscopic and optical properties remain limited.^21,22,25,40,41^ The methoxy group exhibits dual electronic behavior as an electron donor via resonance and an electron-withdrawing group through inductive effects, thereby influencing the electronic characteristics of the imine linkage bridging different aromatic groups.^42–44^ These effects depend strongly on substituent orientation relative to the imine bond.^45^ Ortho-methoxy substitution induces significant perturbations in molecular planarity, dipole moment orientation, π-electron distribution, and intramolecular hydrogen bonding, leading to greater modulation of transition energies than *meta*- and *para*-substitution. In contrast, *para*-substitution is dominated by resonance donation, whereas the *meta* isomer exhibits comparatively weak donor and acceptor behavior through both resonance and inductive effects. Collectively, these trends highlight the role of substitutional orientation in governing the electrodynamics and photophysical characteristics of regioisomeric systems.^20–22,34,35,46^

A detailed understanding of how substituent position governs electronic structure and optical signatures is therefore essential for the rational design of Schiff base chromophores with tailored photophysical properties. In this study, three methoxy-substituted *N*-benzylideneanilines of the type MeOC_6_H_4_–CH=N–C_6_H_4_–*p*-Me (**1**–**3**), synthesized from *p*-toluidine and *o*-, *m*-, and *p*-anisaldehydes, are investigated alongside their unsubstituted analogues **4** (without methoxy substitution) and **5** (lacking both methoxy and methyl groups) (Chart [Fig cht1-fo]). The methoxy group, particularly at the *ortho* position in isomer **1**, may significantly alter the electronic environment of the imine linkage via through-space effects. The influence of methyl substitution on the aniline ring in **1–**-**4** is evaluated for comparison with the Schiff base **5**. Comprehensive spectroscopic, UV–Visible absorption, and DFT/TD-DFT studies are employed to assess the positional effects of methoxy substitution on the electronic structure, photophysical behavior, and nonlinear optical responses of Schiff bases **1**–**5**.

**1 cht1-fo:**
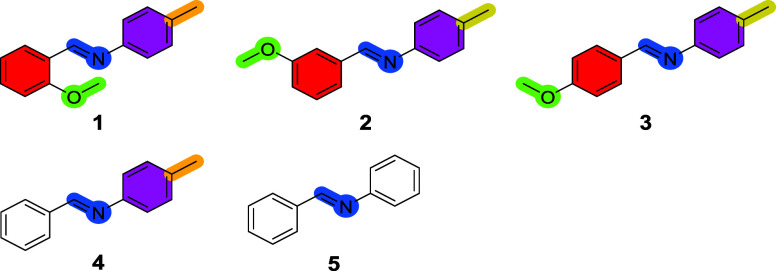
Methoxy-Substituted Schiff Bases **(1**–**3**) and Their Unsubstituted Analogues **4** and **5**.

Density functional theory (DFT), together with its time-dependent extension (TD-DFT), provides a robust basis for interpreting structure–property relationships and extends to the excited-state regime, allowing quantitative simulation of electronic transitions and mechanistic insight into the roles of molecular architecture, substituents, and electron delocalization in optical responses.^47–50^ Solvent effects are treated using the polarizable continuum model (PCM), enabling the prediction of solvatochromic shifts arising from solute–solvent polarization and specific interactions.^24,34–39,51,52^ Combined TD-DFT/PCM analysis supports experimental spectroscopic observations and facilitates the systematic evaluation of methoxy substitution and its positional effects on chromophoric responses in Schiff bases.^36,37,48,53^

## 2 Results and Discussion

### 2.1 Optimized Geometry

The optimized molecular geometries of compounds **1**–**5** are depicted in Figure 1 and the structural parameters are listed in Tables S1–S7. A comparative evaluation of the DFT-optimized geometries of compounds **1**–**5** reveals a high degree of structural consistency across the aromatic frameworks. The aromatic C–C bond lengths lie within the 1.388–1.404 Å range for all five compounds, confirming that the different substituent patterns do not significantly perturb the *π*-electron distribution of the aromatic core. The most electronically diagnostic feature is the imine C=N bond length (R13 in **1**–**3** and R14 in **4** and **5**) which falls within the 1.276–1.278 Å range (Figure S1) demonstrating partial double-bond character.^42,43,54^ The C–Ar′ and N–Ar bond lengths lie in the 1.40–1.47 Å range which reflects subtle resonance shifts induced by the contributing resonance structures that place additional π-density onto the imine carbon shortening the C–Ar′ bond and lengthening the N–Ar bond. Compounds **4** and **5** display slightly shorter C–Ar′ bond lengths indicating enhanced conjugation imposed by the uninterrupted planarity of the aromatic ring without the methoxy group. The aromatic C–H bond lengths remain nearly constant in the range of 1.083–1.086 Å as reported in the literature.^42,43,54^

**Figure 1:**
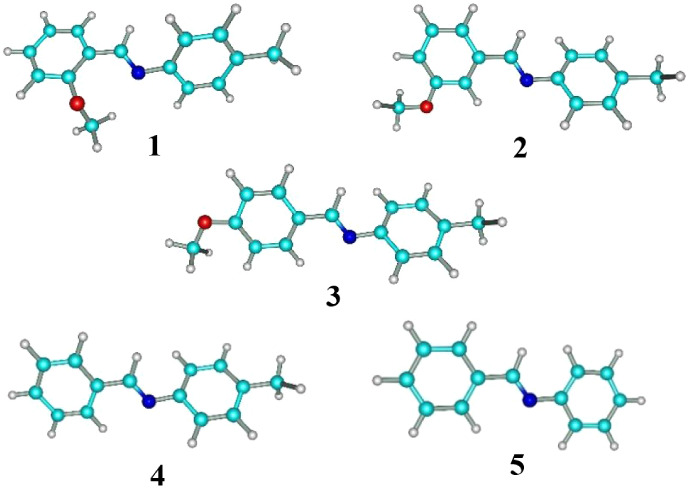
Optimized geometries of the Schiff bases **1**–**5**.

The bond angles in compounds **1**–**5** are dominated by the planar azomethine (C=N) linkage and the adjoining benzylidene and aniline rings. Within the aromatic rings, the internal C–C–C angles exhibit minimal variation within 118–122°.^42,43,54^ This consistency indicates that neither the methoxy nor the methyl substituent significantly perturbs aromatic planarity. The C=C–OMe bond angles in the methoxy-substituted compounds **1**–**3** remain within the typical range for planar conjugated systems and do not disrupt overall electronic communication across the molecules.^42,43,54^ The DFT-optimized geometries reveal that the Schiff bases **1**–**5** maintain an essentially planar C_Ar′_–C=N–C_Ar_ conjugated backbone with uninterrupted conjugation across the C=N unit throughout the series. Thus, positional effects do not arise from backbone distortion, but from the orientation of the methoxy group relative to the phenyl ring.

### 2.2 Molecular Electrostatic Potential (MEP) Mapping

The DFT-generated MEP profiles of the Schiff bases **1**–**5** (Figure 2) illustrate how methoxy and methyl groups on the benzylidene and aniline rings modulate electron distribution which affects chromism, coordination behavior, and molecular polarity.^23,26,27^ Specifically, the electron-donating methoxy substituent in the regioisomeric Schiff bases **1**–**3** leads to enhanced surface polarization with pronounced negative potential at the imine nitrogen and oxygen atoms (red regions, Figure 2), whereas compounds **4** and **5** exhibit comparatively less polarized MEP surfaces due to the weaker electron-donating nature of the methyl substituent and hydrogen.

**Figure 2:**
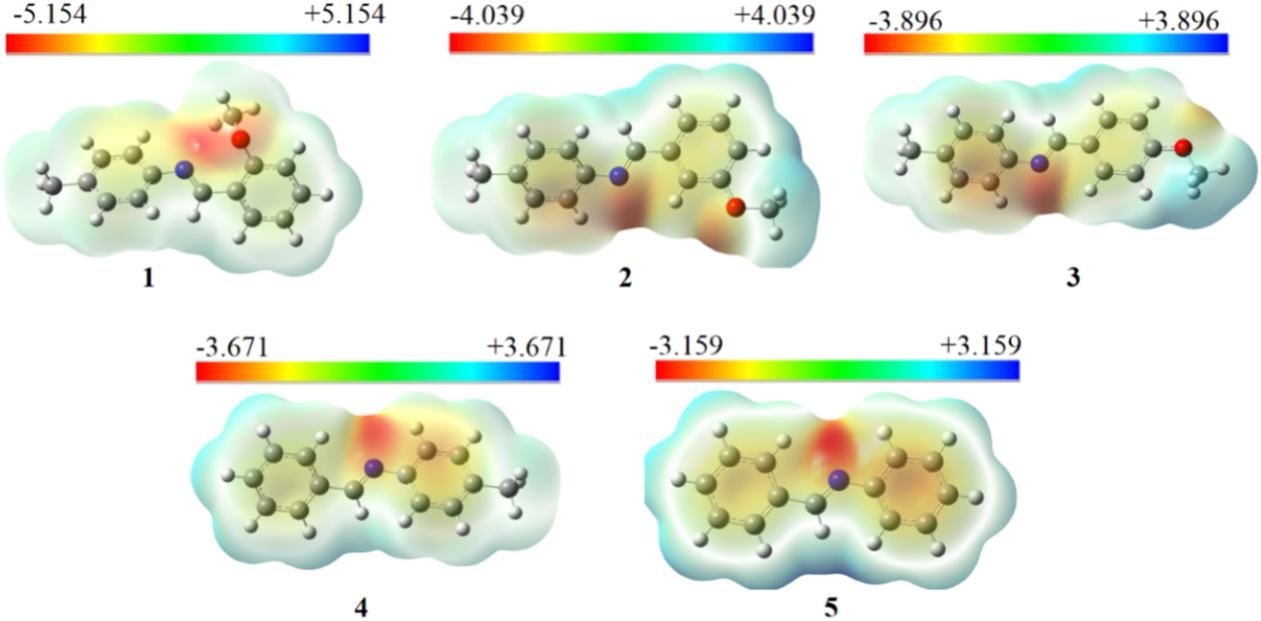
MEP surfaces of the Schiff bases **1**–**5**, highlighting high (red) and low (blue) electron-density regions due to substituent-dependent polarization of the imine core.

The methoxy-substituted regioisomers **1**–**3** display closely related, but diagnostically distinguishable potential surfaces. In each case, the methoxy substituent functions as a strong +M donor increasing electron density on the substituted region of the benzylidene ring. This results in a pronounced π-cloud anisotropy depending on the orientation of the methoxy group relative to the imine axis: (i) *ortho*-substitution induces modest torsion and a localized negative potential due to proximal lone-pair conjugation; (ii) *meta*-substitution suppresses resonance transmission resulting in a less pronounced negative (red) region (Figure 2); and (iii) *para*-substitution enables maximal π-conjugation with the C=N unit yielding the most extended negative potential across the benzylidene framework. Across the regioisomers **1**–**3**, the *para*-methyl group on the aniline ring acts as a weak electron donor, thereby increasing electron density on the aniline segment and slightly reducing the electrophilicity of the imine carbon. The Schiff bases **4** and **5**, which differ from the substitution patterns of **1**–**3**, display correspondingly distinct MEP topologies. The absence of an electron-donating methoxy group in **4** and **5** reduces the extent of π-delocalization onto the benzylidene ring, localizing the negative potential to the immediate vicinity of the imine nitrogen rather than transmitting across the molecular framework.

### 2.3 FT-IR Spectra of Schiff Bases **1**–**5**

The FT-IR spectra of the Schiff bases **1**–**5** are presented in Figures S2 and S3. The key vibrational assignments along with their experimental and DFT-calculated wavenumbers are presented in Table S8. The FT-IR spectral study demonstrates that the position of the methoxy substituent exerts a significant influence on the prominent IR vibrational modes with the azomethine ν­(C=N) vibration being the most affected (Figure 3). The ν­(C=N) band appears at the highest frequency for the *para*-isomer **3** (1659 cm^–1^) and at the lowest frequency for the *ortho*-isomer **1** (1632 cm^–1^), whereas the remaining compounds display this band at 1627 cm^–1^.^55^ The DFT-scaled ν­(C=N) and ν­(H–C) vibrational frequencies of compounds **1**–**5** show good agreement with experimental values with a mean |Δ_exp–DFT_| of 21.72 cm^–1^ and 14.52 cm^–1^, respectively (Figure 3).

**Figure 3:**
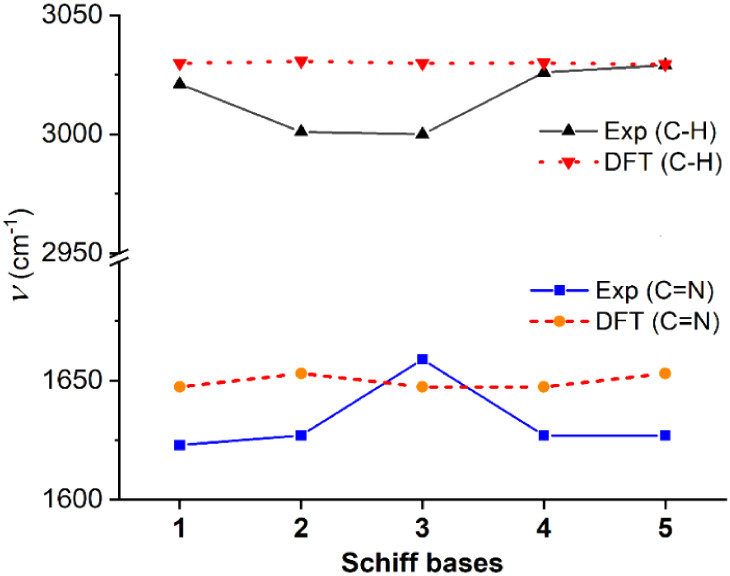
Experimental and DFT frequencies for ν­(C=N) and ν­(C–H) vibrations of **1**–**5**.

The ν­(O–C) vibration further reflects this trend with a higher frequency for the *ortho*-isomer **1** and relatively lower frequencies for the *meta*- and *para*-isomers **2** and **3** indicating differences in resonance delocalization in **1**–**3**. The aliphatic O–C stretching vibration of the methoxy group occurs at 1271 cm^–1^ for the *meta*-isomer **2**, a blue shift of 23.8 cm^–1^ compared to the average of its *ortho*- and *para*-isomers (1247.25 cm^–1^). DFT locates this vibration within a narrow window of 1248–1250 cm^–1^ with a mean |Δ_exp–DFT_| value of 8.0 cm^–1^. The Ar–O–C stretching vibration of the methoxy group appears at 1043 cm^–1^ for the *ortho*- and *meta*-isomers which is higher than that of the *para*-isomer by 15.5 cm^–1^ (1027.5 cm^–1^), whereas the DFT computed values appear at 1018–1022 cm^–1^ with the mean |Δ_exp–DFT_| value of 10.66 cm^–1^. The aromatic ring-based vibrational modes remain largely unchanged, confirming that the electronic effect is localized primarily at the benzylidene–imine segment. The benzylidene ring C=C_Ar_ stretching vibration shows modest, while aniline-ring CH and C–N stretching vibrations remain essentially invariant indicating limited transmission of substituent effects across the imine linkage.

### 2.4 ^1^H and ^13^C NMR Spectra

The ^1^H and ^13^C NMR spectra of the Schiff bases **1**–**4** are presented in the Supporting Information (Figures S4–S7). The experimental chemical shift values are compared with the corresponding DFT-calculated NMR chemical shifts (Tables S9 and S10).

#### 2.4.1 ^1^H NMR Spectra

The azomethine (C ***H*** =N) protons of the Schiff bases **1**–**5** resonate within a narrow chemical shift value of δ = 8.4–8.9 ppm.^56–59^ Among the methoxy isomers, the *ortho*-isomer **1** shows the most downfield shift (δ = 8.9 ppm) indicating pronounced deshielding arising from induced anisotropic and intramolecular electronic effects associated with the *ortho*-methoxy group.^40^ The *meta*- and *para*-isomers **2** and **3** resonate at δ = 8.4 and 8.6 ppm, respectively, demonstrating reduced and position-dependent resonance contributions from the methoxy substituent. Compounds **4** and **5** exhibit similar chemical shifts (δ = 8.6 and 8.6 ppm). The corresponding DFT-predicted chemical shifts cluster within a narrow range of δ = 8.5–8.6 ppm for **1**–**4**, whereas compound **5** shows a significantly downfield value (δ = 9.4 ppm) reflecting enhanced polarization of the azomethine proton in the absence of electron-donating methoxy and methyl substituents. The trend in the experimental and DFT-calculated ^1^H chemical shifts of the azomethine protons for compounds **1**–**5** is illustrated in Figure 4a.

**Figure 4:**
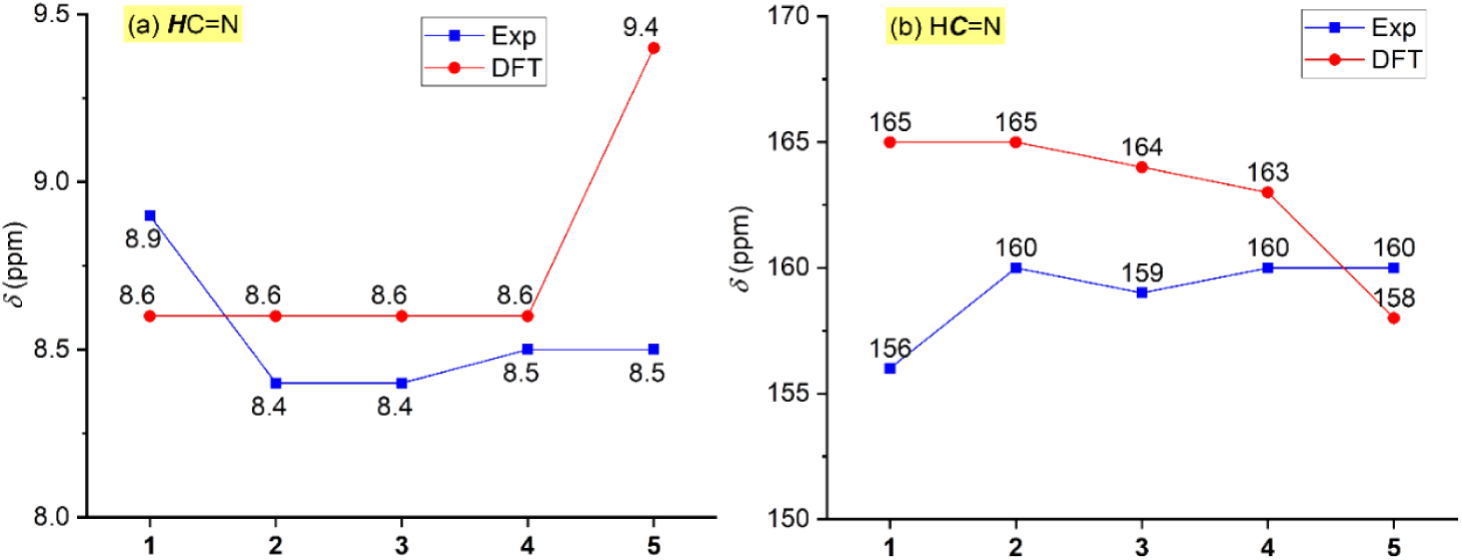
Experimental and DFT-calculated NMR chemical shifts of the azomethine protons (a) and carbons (b) of Schiff bases **1**–**5**.

The chemical shift values of the methyl protons on the aniline ring (Ar) in **1**–**4** are identical (δ = 2.4 ppm) falling within the region typical of aromatic methyl protons (δ = 2.2–2.5 ppm).^56–58^ Similarly, the methoxy protons in the isomers **1**–**3** resonate consistently at δ = 3.89 ppm, a value typical of aromatic methoxy protons (δ = 3.7–4.0 ppm).^40^ This uniformity in the chemical shift values suggests that the positional electronic effects are localized and primarily influence the azomethine (C ***H*** =N) proton, while the methyl and methoxy substituents experience comparatively negligible variations in their electronic environments. The methoxy substituent renders an increased electron density across the benzylidene ring (Ar′) as evidenced by the upfield chemical shift values of the benzylidene ring protons of the Schiff bases **1**–**3** relative to that of **4** (δ = 7.2–7.9 ppm) and **5** (δ = 7.5–7.9 ppm) which lack the methoxy group. The ^1^H NMR spectra of **1** and **2** show four aromatic proton resonances in the range δ = 7.0–8.1 and δ = 7.2–7.4 ppm, respectively, while **3** exhibits two aromatic resonances at δ = 7.0 and 7.8 ppm. The DFT-calculated chemical shift values for the aromatic protons are consistent with the experimental values. The aniline rings of the Schiff bases **1**–**4** are in a 1,4-disubstituted pattern, and therefore their ^1^H NMR spectra contain two aromatic proton resonances in the range δ = 7.1–7.2 ppm. In **5**, where there is no methyl group on the aniline ring, these proton resonances appear at δ = 7.4 ppm. The methoxy substituent on the benzylidene ring does not produce a noticeable influence on the electronic environment of the aniline ring confirming that substituent effects are not significantly transmitted across the imine linkage.^56–59^

#### 2.4.2 ^13^C NMR Spectra

The ^13^C chemical shifts of the azomethine (***C*** H=N) carbons in the Schiff bases **1**–**5** are more influenced by the combined effects of substituent-dependent resonance interactions, inductive effects, and steric perturbations transmitted through the aromatic framework than by azomethine protons.^60,61^ Notably, the *ortho*-isomer **1** exhibits a pronounced upfield experimental chemical shift value of δ = 156 ppm compared to other isomers which show values of δ = 159 ppm (**3**) and 160 ppm (**4**). This upfield shift is attributed to efficient π-electron donation (+R effect) by the *ortho*-methoxy substituent to the C=N moiety. In contrast, the *meta*- and *para*-methoxy groups in **2** and **3** transmit comparatively weaker electronic effects to the azomethine carbon, resulting in reduced shielding and downfield chemical shifts of δ = 160 and 159 ppm, respectively.^25,40^ The *para*-methyl substituent in **4** contributes only a weak +I inductive effect from the aniline ring, providing limited π-electron delocalization onto the C=N framework and exhibiting a downfield shift. Compound **5**, which lacks both methoxy and methyl substituents, is governed primarily by the intrinsic polarization of the C=N bond leading to the most deshielded azomethine carbon resonance at δ = 160 ppm. The negligible variation in the DFT-predicted ^13^C chemical shifts of the azomethine carbon of **1**–**3** (δ = 165, 165, and 164 ppm, respectively) together with slightly upfield chemical shifts calculated for **4** (δ = 163 ppm) and **5** (δ = 158 ppm) underscores the limitations of the computational model in reproducing the subtle steric and anisotropic effects observed experimentally (Figure 4b).

The carbon directly bonded to the azomethine carbon (***C***_Ar′_) exhibits pronounced substituent sensitivity. Experimentally, the ***C***_Ar′_ carbons in compounds **1** and **3**, bearing *ortho*- and *para*-OCH_3_ groups on the benzylidene ring, are highly shielded with chemical shifts of δ = 125 and 129 ppm, respectively. In compound **2**, where the *meta*-OCH_3_ group is incapable of effective resonance interaction, the ***C***_Ar′_ carbon is significantly deshielded (δ = 138 ppm). Compounds **4** and **5**, which lack electron-releasing substituents on this ring, display downfield resonances at δ = 136 ppm, respectively. DFT calculations reproduce the same qualitative trend as illustrated in Figure 5a with upfield values for **1**–**3** relative to **4** and **5**. The theoretical chemical shift values are systematically higher (by 8–12 ppm) than the experimental values; however, the relative substituent-driven trends are well reproduced. The ^13^C chemical shift values of the carbon attached to the imine nitrogen (***C***_Ar_) of the Schiff bases **1**–**4** fall within a narrow range of δ = 149–150 ppm. In contrast, compound **5** which is devoid of a methyl group exhibits a downfield shift at δ = 152 ppm. The DFT-computed chemical shift values for **1**–**5** fall in the range of δ = 154–158 ppm with the most shielded carbon in **5** (δ = 154 ppm) (Figure 5b).

**Figure 5:**
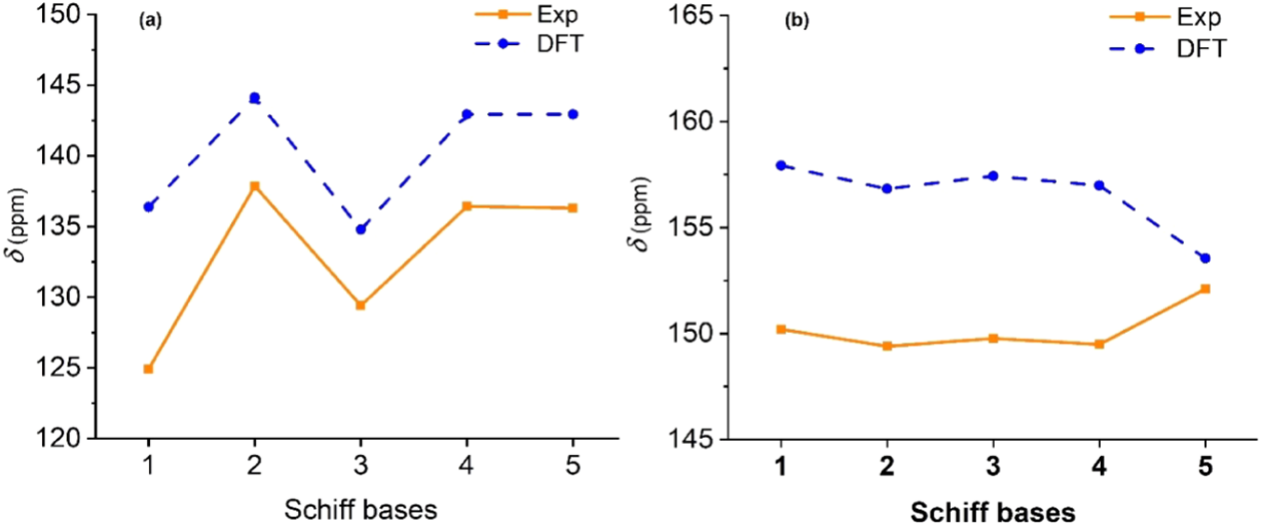
Experimental and DFT-calculated ^13^C NMR chemical shifts for ***C***_Ar′_ (a) and ***C***_Ar_ (b) of Schiff bases **1**–**5**.

The aromatic carbons of the benzylidene ring (Ar′) display large chemical shift variation for the *ortho*-isomer **1** (δ = 111–132 ppm). In contrast, the *para*-isomer **3**, owing to its higher molecular symmetry and more uniform π-electron delocalization, exhibits small aromatic carbon chemical shift variation (δ = 115 and 130 ppm) compared to isomers **1** and **2** (δ = 119–130 ppm). Compound **4** shows comparatively downfield aromatic carbon resonances (δ = 121, 126, and 129 ppm) and **5** exhibits the most downfield shifts (δ = 129, 129, and 131 ppm) due to reduced electron donation and diminished π-conjugation effects with concomitant deshielding of the benzylidene ring. The DFT-calculated benzylidene aromatic carbon chemical shifts qualitatively reproduce the experimental trend for **1** (128–142 ppm), **2** (112–130 ppm), **3** (113–139 ppm), **4** (131–137 ppm), and **5** (131–333 ppm) showing progressively increased deshielding (Figure 5b). Minor quantitative differences between calculated and experimental chemical shift values fall within the typical error range reported for DFT-based ^13^C NMR predictions (∼1–3 ppm).^62^ Such deviations primarily reflect intrinsic functional-dependent shielding approximations and the limitations of the continuum solvation model (PCM), which do not explicitly capture specific solute–solvent interactions or dynamic averaging effects.^52,63^

The aromatic carbons of the aniline ring (Ar) exhibit minimal chemical shift variation across compounds **1**–**4** (δ = 121–131 ppm) indicating limited transmission of substituent effects through the imine linkage. Compound **5** shows slightly shifted aniline carbon resonances (δ = 129–131 ppm). The DFT calculations predict aniline carbon chemical shift values in the range of 116–138 ppm for compounds **1**–**5** in agreement with the experimental values. The methyl carbons on the aniline ring in compounds **1**–**4** resonate at δ = 21 ppm and the methoxy carbon in compounds **1**–**3** resonates at δ = 55–56 ppm. The DFT computations predict chemical shift values of 21–22 ppm for the methyl carbons and 56–64 ppm for the methoxy carbons indicating minimal electronic and positional sensitivity of these substituents. Overall, the *ortho*-methoxy substituent exerts the strongest electronic influence causing pronounced deshielding in both ^1^H and ^13^C environments, whereas the methyl group shows a weaker, localized donor effect. Consequently, benzylidene ring carbons are sensitive to substituent type and position, while aniline ring carbons remain largely unaffected. Thus, the substitution pattern critically controls the electronic environment and NMR properties of Schiff bases **1**–**5**.

### 2.5 UV–Visible Absorption Spectra

The UV–Vis electronic absorption spectra of the Schiff bases **1**–**3** are recorded using solutions of identical concentration (2.50 × 10^–5^ M) in solvents of varying polarity, ranging from *n*-hexane to water. The compound-specific and solvent-specific electronic spectra are presented in Figures 6 and 7, respectively, and the corresponding spectral parameters are summarized in Table S11. For the Schiff bases **1**–**3**, the low-energy absorption band is attributed to the π → π* transition associated with the azomethine (HC=N) chromophore.^41,64,65^ Examination of the λ_max_ values and absorbance (A) of the π → π* transitions of **1**–**3** across the solvent series reveals that the donor strength and degree of conjugation imparted by the methoxy substituent provide a structural basis for the observed solvatochromic behavior.^24,39^

**Figure 6:**
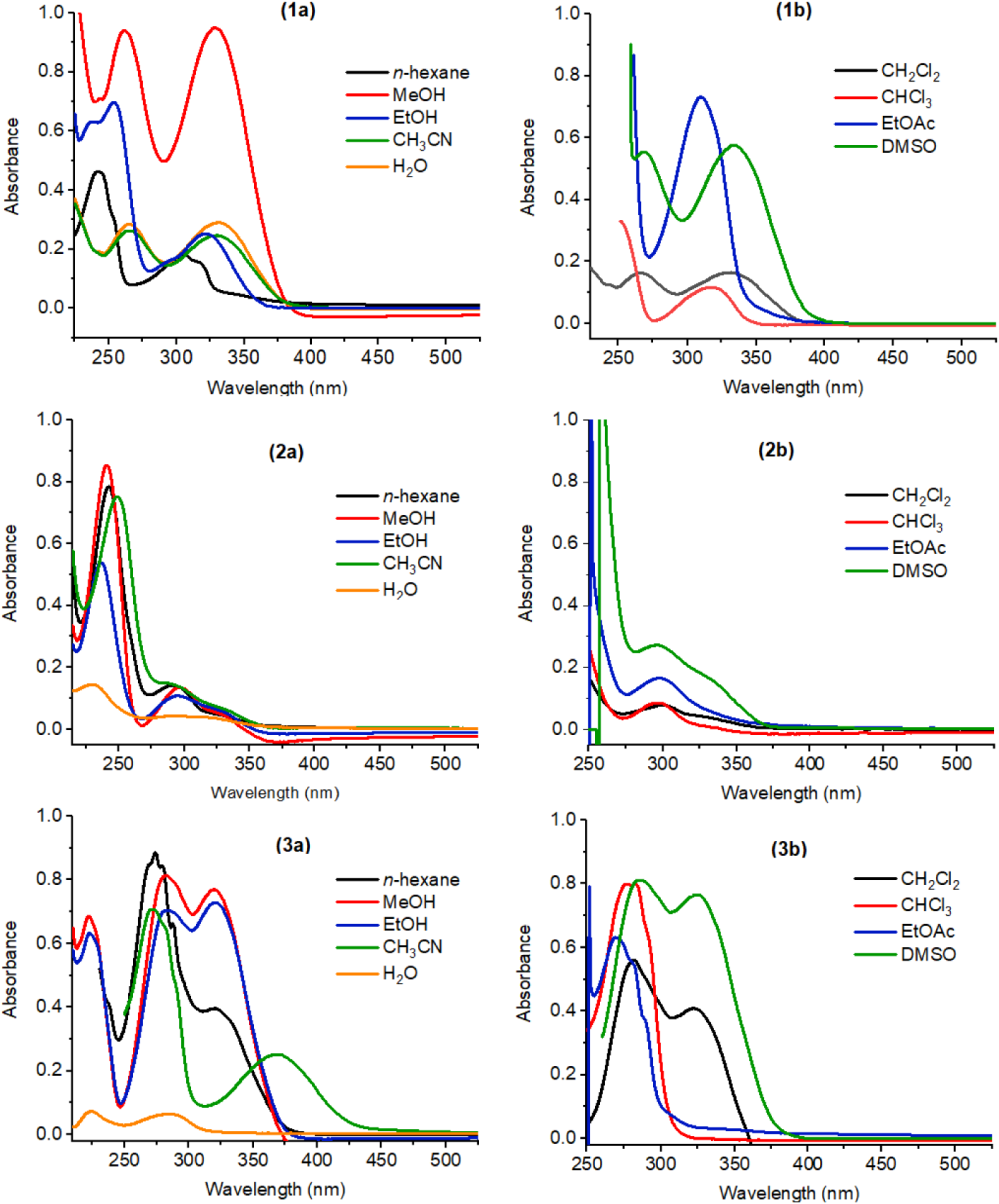
UV–Vis absorption spectra of Schiff bases 1–3 in solvents of increasing polarity, ranging from *n*-hexane to water. Panels **1a** and **1b** for compound **1**, **2a** and **2b** for compound **2**, and **3a** and **3b** for compound **3**. The spectra are separated into solvents with UV–Vis cut-off wavelengths of 190–205 nm (a) and >230 nm (b) to facilitate visualization of absorption bands that are not obscured by solvent absorption.

**Figure 7:**
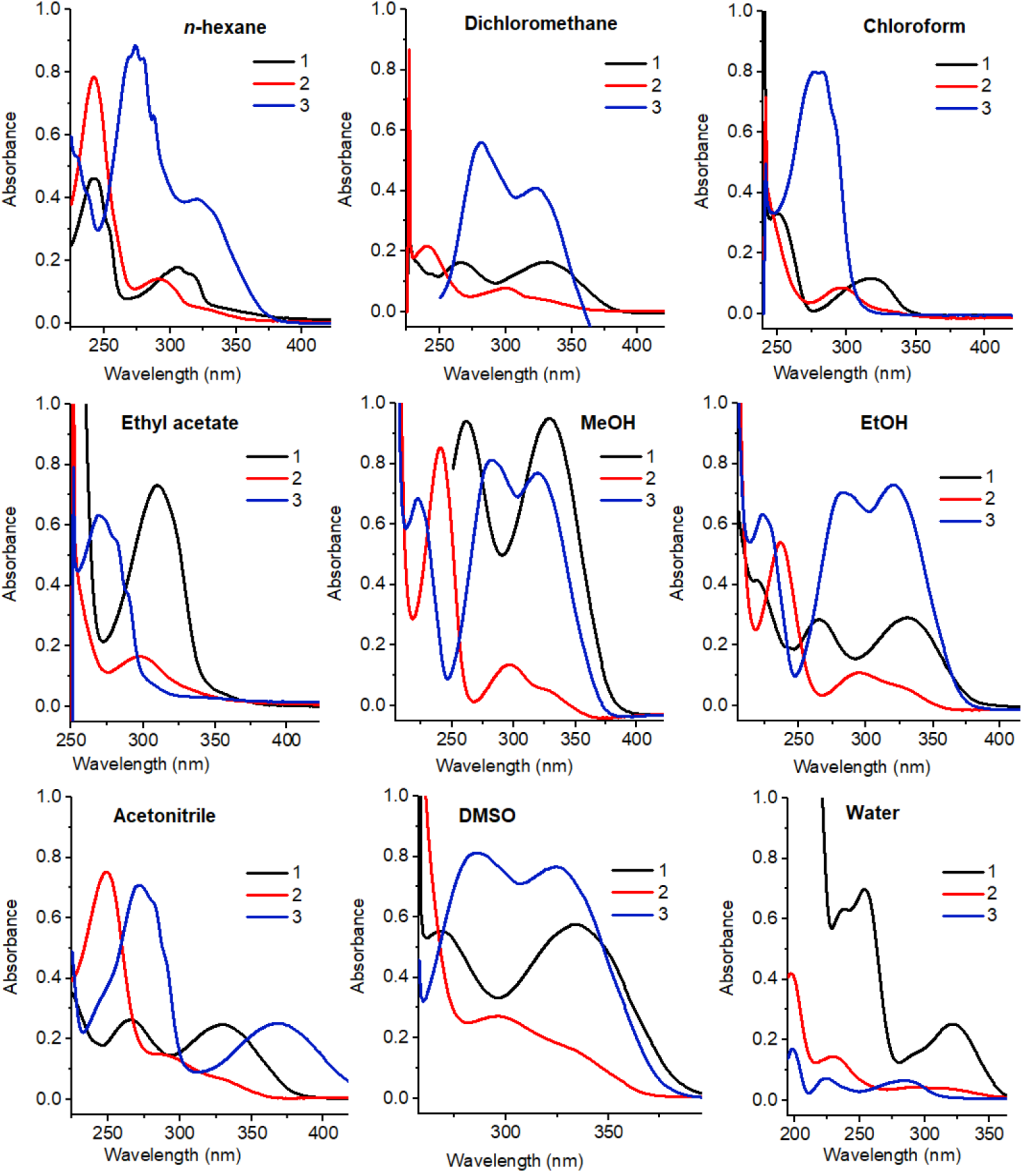
Solvent-specific UV–Vis absorption spectra of Schiff bases **1**–**3**.

#### 2.5.1 Substituent-Dependent Chromism of **1**–**3**

The UV–Vis electronic absorption spectra of the Schiff bases **1**–**3** display pronounced substituent-position-dependent modulation of the imine (C=N) chromophore absorption (Figure 6). In nonpolar *n*-hexane, where intrinsic electronic effects dominate, the λ_max_ value of the π → π* transition follows the trend: **3** (320.8 nm, A = 0.397) > **1** (305.9 nm, A = 0.180) ≫ **2** (289.8 nm, A = 0.142), reflecting strong +M donation from the *para*-OCH_3_ group in **3**, sterically attenuated resonance interaction in the *ortho*-isomer **1,** and no conjugation in the *meta*-isomer **2**. The *para*-derivative **3** exhibits a red-shifted, high-intensity band in moderately polar solvents (e.g., MeOH and EtOH), while the *ortho*-isomer **1** shows more intense bands in polar media. In contrast, the *meta*-isomer **2** remains largely hypsochromic (λ_max_ = 294–299 nm) in most solvents and exhibits a weak low-energy band in highly polar aprotic media.

#### 2.5.2 Solvatochromism and UV–Visible Spectra of 1–3: *Nonpolar and Moderately Polar Solvents*

Systematic variation of the solvent environment from *n*-hexane to water displays pronounced and compound-specific solvatochromic shifts in the UV–Vis spectra (Figure 7).^36–39^ The absorption band of compound **1** undergoes a substantial red-shift from 305.9 nm in *n*-hexane to 331.9 nm in DCM (A = 0.088) and 322.0 nm in chloroform (A = 0.927). Compound **3** exhibits a highly intense band at 282.4 nm (A = 0.800) in DCM, although the wavelength shift is modest relative to that in *n*-hexane, while compound **2** exhibits only minor shifts (λ_*max*_ = 299–300 nm). In dichloromethane and chloroform, the *ortho*- and *para*-isomers **1** and **3** exhibit positive solvatochromism, whereas the *meta*-isomer **2** remains relatively insensitive. These results confirm that in nonhydrogen-bonding media, solvatochromic behavior is primarily governed by substituent position and solvent polarity.

##### 2.5.2.1 Dipolar and Protic Solvents

Hydrogen bonding and dipolar solvation produce enhanced substituent differentiation in the electronic spectra of these compounds. In ethyl acetate, compound **1** absorbs at 309.6 nm (A = 0.731) compared with 298.5 nm (A = 0.166) for **2** and 268.8 nm (A = 0.632) for **3**. The ICT band for compound **1** is red-shifted to 328.2 nm (A = 0.950) in MeOH and 330.7 nm (A = 0.291) in EtOH, while compound **3** exhibits the ICT band at 319.5 nm (A = 0.769) in methanol and at 320.8 nm (A = 0.729) in EtOH, and for **2**, it remains largely unshifted (298.5 nm in ethyl acetate, 296.0 nm in MeOH, and 294.6 nm in EtOH), reflecting minimal polarizability of its excited state.

##### 2.5.2.2 Highly Polar Solvents

The most dramatic solvent polarity-dependent shift of the ICT absorption bands of **1**–**3** occurs in acetonitrile and dimethyl sulfoxide. The ICT band for **1** appears at 330.7 nm (A = 0.247) in MeCN and 333.3 nm (A = 0.575) in DMSO, while compound **3** exhibits a significant red-shift to 369.1 nm (A = 0.253) in MeCN. The *meta*-isomer **2** exhibits unusually long-wavelength, low-intensity absorption bands at 397.5 and 386.3 nm in MeCN and DMSO, respectively. In water, specific solute–solvent interactions dominate over the ICT transition and consequently compound **1** exhibits the ICT absorption at 320.8 nm (A = 0.252), while for compound **2** this absorption band is blue-shifted to 293.5 nm (A = 0.044). The *para*-isomer **3** exhibits a pronounced hypsochromic shift at 284.8 nm (A = 0.525) indicating that hydrogen bonding in water stabilizes the ground state with a concomitant lowering of the ICT character.

#### 2.5.3 TD-DFT-Simulated UV–Vis Spectra

The UV–Vis absorption spectra of the Schiff bases **1**–**5** are computed in the gas phase and in chloroform, ethanol, and acetonitrile using TD-DFT at the B3LYP-6311++G­(d,p) level of theory with the IEFPCM solvation model.^67^ The simulated spectra are shown in Figure 8 (compound-specific) and Figure 9 (solvent-specific) and the calculated absorption maxima (λ_max_) and ε_max_ values are summarized in Table S12. In the gas phase, **1**–**5** exhibit two principal absorption bands. The high-energy band at 274–293 nm is assigned to the π → π* transition of the delocalized aromatic–azomethine π-conjugated framework, whereas the low-energy band at 317–347 nm is attributed predominantly to the *n* → π* transition involving the azomethine nitrogen lone pair. Compounds **1**, **2**, and **4** exhibit both the π → π* and *n* → π* transitions in a very narrow wavelength range of 274–275 nm and 345–347 nm, respectively, while the absorption of **3** is red-shifted (292.8 and 338.2 nm) with higher intensity. Compound **5** displays the *n* → π* transition relatively at lower energy (317.7 nm) with high intensity (ε = 32,915 M^–1^ cm^–1^) reflecting substituent-dependent modulation of the excited-state energies. Upon solvent inclusion, these transitions undergo varying degrees of bathochromic shift and intensity modulation depending on substituent identity and solvent polarity.

**Figure 8:**
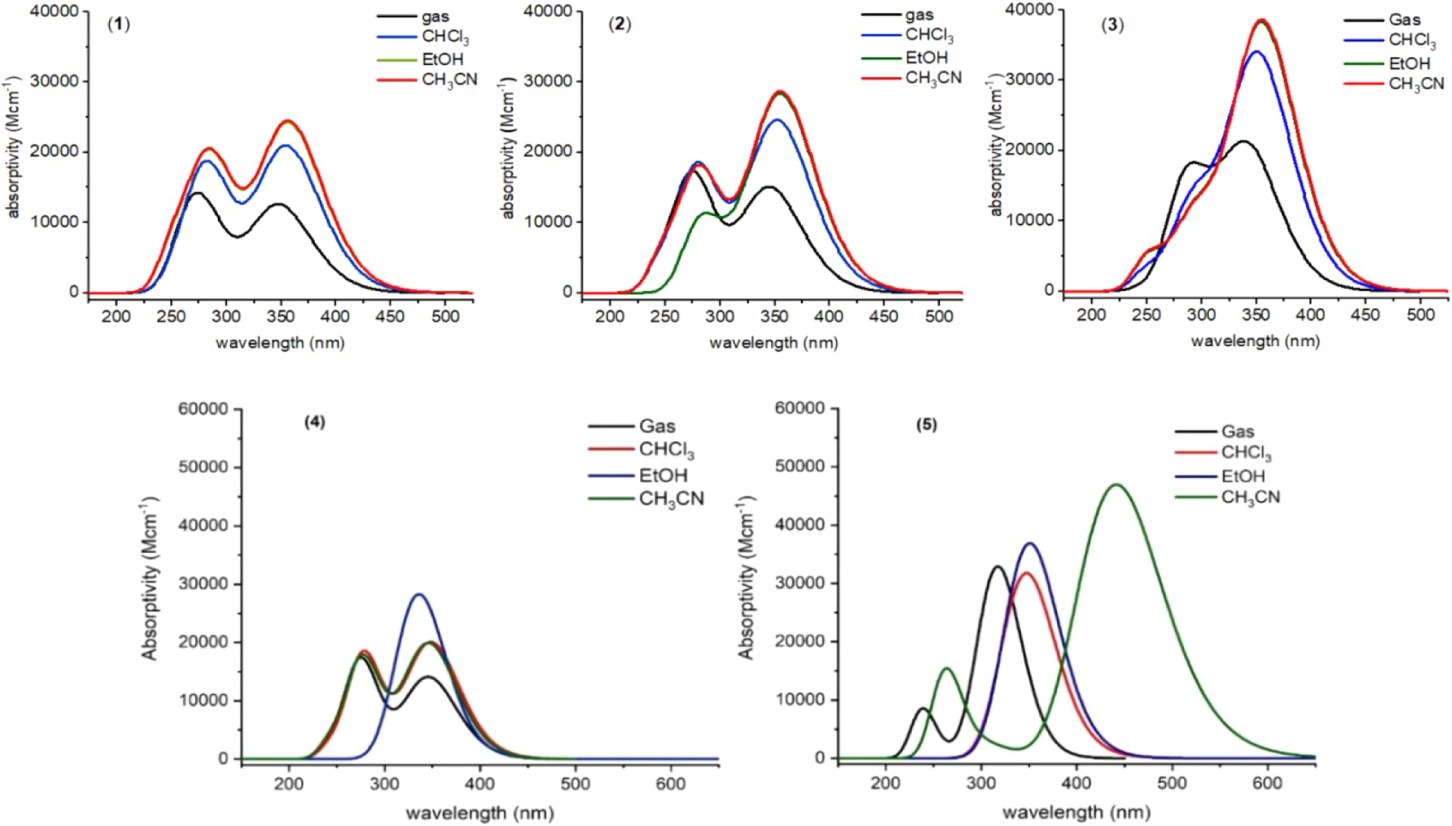
Compound-specific TD-DFT-simulated UV–Vis absorption spectra of **1**–**5**.

**Figure 9:**
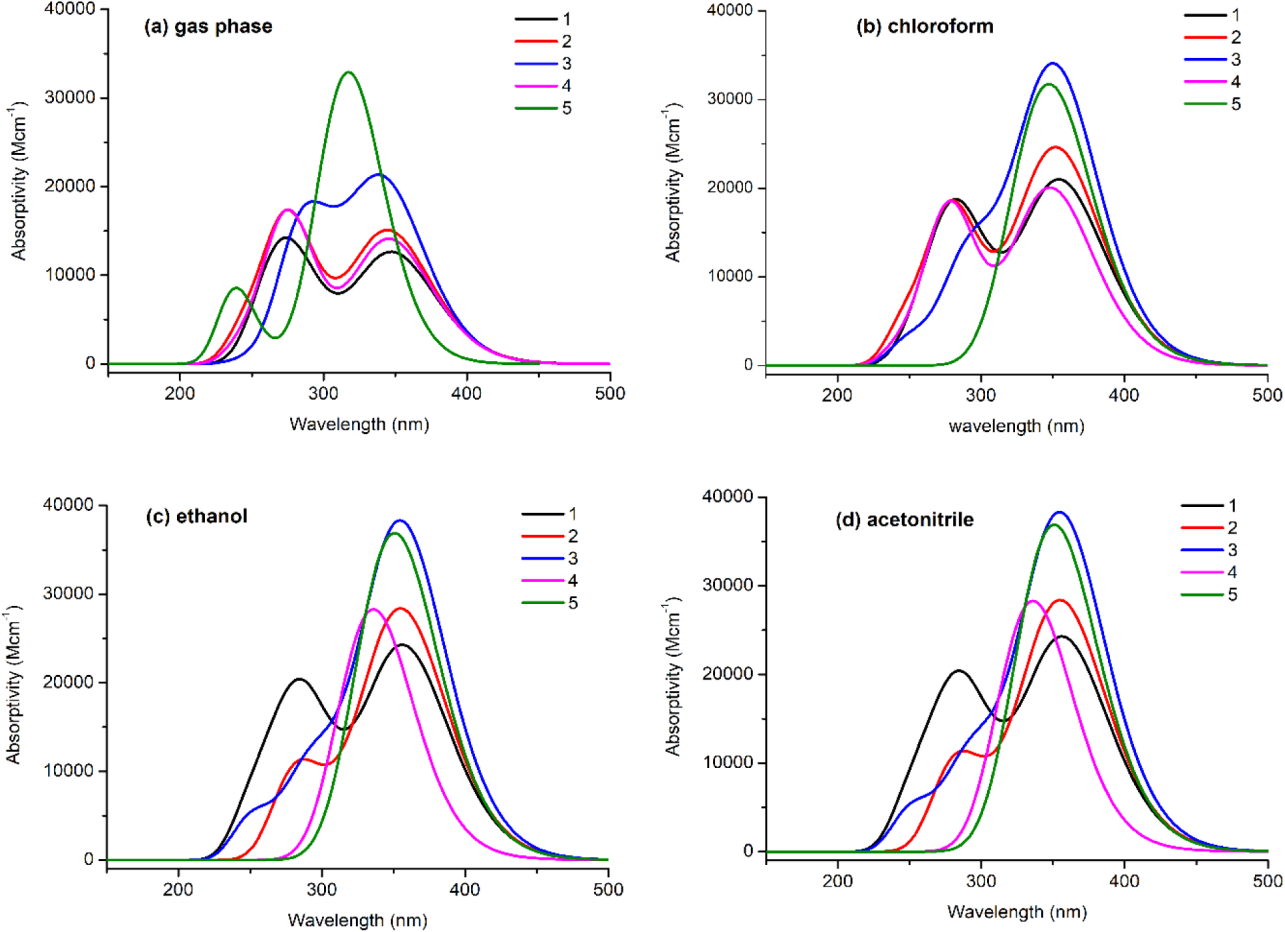
Solvent-specific TD-DFT-simulated UV–Vis absorption spectra of **1**–**5:** (a) gas phase, (b) chloroform, (c) ethanol, and (d) acetonitrile.

The *π* → π* band for compound **1** shifts from 274.4 nm (*ε* = 14,248 M^–1^ cm^–1^) in the gas phase to 284.5 nm (ε = 20,425 M^–1^ cm^–1^) in ethanol and 284.5 nm (*ε* = 20,580 M^–1^ cm^–1^) in acetonitrile indicating a moderate bathochromic shift accompanied by a significant increase in molar absorptivity. This moderate bathochromic shift and intensity enhancement indicate stabilization of a polar excited state in solvent media. The n → π* band also undergoes a more pronounced red-shift from 347.2 nm (*ε* = 12,649 M^–1^ cm^–1^) in the gas phase to 356.5 nm (*ε* = 24,542 M^–1^ cm^–1^) in acetonitrile, reflecting significant solvent stabilization of the lone-pair-based excited state. Compound **2** displays a comparable, but slightly less regular solvatochromic response. The *π* → π* transition shifts from 275.2 nm in the gas phase to 287.2 nm in ethanol with ε values in the 17,386–18,785 M^–1^ cm^–1^ range indicating modest solvent sensitivity. In contrast, the n → π* band exhibits a consistent bathochromic shift from 344.8 nm (*ε* = 15,100 M^–1^ cm^–1^) in the gas phase to 354.7 nm (*ε* = 28,719 M^–1^ cm^–1^) in acetonitrile accompanied by a substantial increase in intensity. Compound **3** exhibits pronounced solvatochromism with the higher-energy *π* → π* band shifting from 292.8 nm (*ε* = 18,342 M^–1^ cm^–1^) in the gas phase to 350.4 nm (*ε* = 34,099 M^–1^ cm^–1^) in chloroform and 354.7 nm (*ε* = 38,322–38,653 M^–1^ cm^–1^) in ethanol and acetonitrile reflecting a substantial bathochromic shift accompanied by marked intensity enhancement. The low-energy n → π* band similarly shifts from 338.2 nm (*ε* = 21,352 M^–1^ cm^–1^) in the gas phase to 350.4–354.7 nm in solution with significantly higher ε values indicating increased excited-state delocalization in polar media.

In contrast, compound **4** retains two well-resolved absorption bands with minimal wavelength dispersion across solvent media. The high-energy band shifts slightly from 275.2 nm (*ε* = 17,242 M^–1^ cm^–1^) in the gas phase to 278.4 nm (*ε* = 18,574 M^–1^ cm^–1^) in chloroform and 277.6 nm (*ε* = 17,944 M^–1^ cm^–1^) in acetonitrile indicating negligible solvent-induced shift of the absorption bands. The low-energy band which appears at 345.6 nm (*ε* = 14,121 M^–1^ cm^–1^) in the gas phase is shifted to 348.0 nm (*ε* = 20,085 M^–1^ cm^–1^) in chloroform, 336.0 nm (*ε* = 28,306 M^–1^ cm^–1^) in ethanol, and 345.6 nm (*ε* = 19,943 M^–1^ cm^–1^) in acetonitrile. Thus, the variation in *λ*_max_ remains relatively small and nonsystematic, while the molar absorptivity shows more pronounced solvent-dependent modulation, particularly in ethanol where ε increases substantially. This indicates that compound **4** exhibits limited solvatochromism in terms of band position, but moderate solvent influence on transition intensity suggesting restricted excited-state polarization. Compound **5** shows contrasting behavior where the high-energy π → π* band undergoes a hypsochromic shift from 293.3 nm (*ε* = 8572 M^–1^ cm^–1^) in the gas phase to 263.2 nm (*ε* = 15,425 M^–1^ cm^–1^) in acetonitrile, whereas the lower-energy n → π* band shifts markedly from 317.7 nm (*ε* = 32,915 M^–1^ cm^–1^) to 348.0–350.0 nm in chloroform and ethanol and further to 441.0 nm (*ε* = 46,957 M^–1^ cm^–1^) in acetonitrile indicating pronounced stabilization of a highly polar charge-transfer-type excited state in a strongly polar medium. Thus, TD-DFT computation indicates that solvent polarity modulates spectral shifts with solvatochromic responses governed by substituent electronic effects.

#### 2.5.4 Correlation of λ_max_ with Reichardt Solvent Polarity Parameter *E*_T_(30)

The Reichardt plot based on the solvatochromic behavior of Reichardt’s dye Betaine 30 is used to study the influence of solvent polarity on the intramolecular charge transfer of a molecule.^66^ The dependence of λ_max_ on *E*_T_(30) is shown in Figure 10. The TD-DFT captures the experimentally observed positive solvatochromic trend for compound **1**, although the magnitude of the slope (0.452 ± 0.131) is underestimated relative to the experimental value (1.222 ± 0.315). In the case of compound **2**, both experiment and theory show near-zero slope (0.032 ± 0.440 experimental and 0.479 ± 0.144 computed) confirming weak solvent response for the *meta*-methoxy derivative. Larger deviations between experimental and calculated solvatochromic parameters are observed for compounds **3**–**5**. The TD-DFT predicts a moderate positive E_*T*_(30) slope of 0.796 ± 0.244 for **3**, but the experimental slope of 1.181 ± 2.665 is associated with uncertainty, limiting a definitive assessment of solvent polarity dependence on the Reichardt solvent polarity parameter *E*_T_(30). In the case of compound **4,** TD-DFT yields a small negative slope of −0.424 ± 0.292 and the experimental *λ*_max_ values display a bathochromic shift across the solvent series (slope = 1.214 ± 1.583). Similarly, for compound **5**, TD-DFT predicts enhanced solvent sensitivity as indicated by a large positive slope of 2.882 ± 3.663, whereas the experimental data reveals inverse solvatochromism (−1.076 ± 0.727) suggesting solvent-dependent ground-state stabilization and conformational effects.

**Figure 10:**
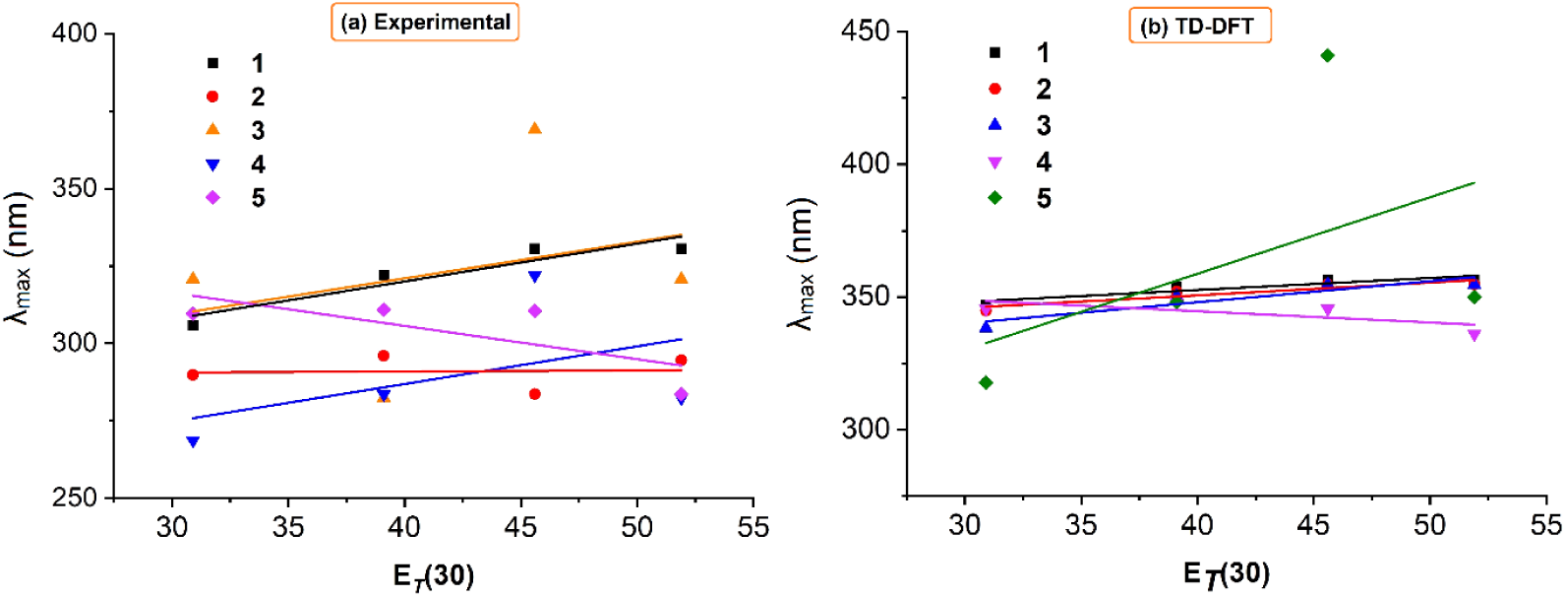
Linear correlation plots of λ_max_ vs E*_T_* (30): (a) experimental and (b) TD-DFT-computed UV–Vis absorption spectra of Schiff bases **1**–**5**.

### 2.6 Mulliken Population Analysis

The DFT-calculated Mulliken charges of atoms **1**–**5** are given in Tables S1–S5. The charges on the azomethine H, C, and N atoms, adjoining aromatic carbons (**C**_Ar′_ and **C**_Ar_), substituent-centered atoms (**C**OMe, **O**Me, **C**Me), and carbons of O**C**H_3_ and **C**H_3_ substituents are summarized in Table S13, and the trend in the charges of the azomethine atoms is depicted in Figure 11. The azomethine carbon (**C**=N) exhibits the highest Mulliken atomic charge variation across the series identifying it as the principal site of substituent-induced polarization. The high positive Mulliken value of the azomethine carbon in **1** reflects charge withdrawal dictated by the methoxy orientation, whereas the negative values for **2** and **4** indicate enhanced electron delocalization toward the imine carbon. The very low negative charge of azomethine C in **3** arises from partial compensation between inductive and resonance effects. In the unsubstituted analogue **5**, the weak negative charge of the azomethine carbon confirms suppression of donor-assisted polarization. In contrast, the azomethine nitrogen (C=**N**) shows only modest charge variation (0.156–0.242) and the azomethine hydrogen remains nearly invariant (0.067–0.092), confirming that substituent effects primarily perturb the carbon center of the imine linkage.

**Figure 11:**
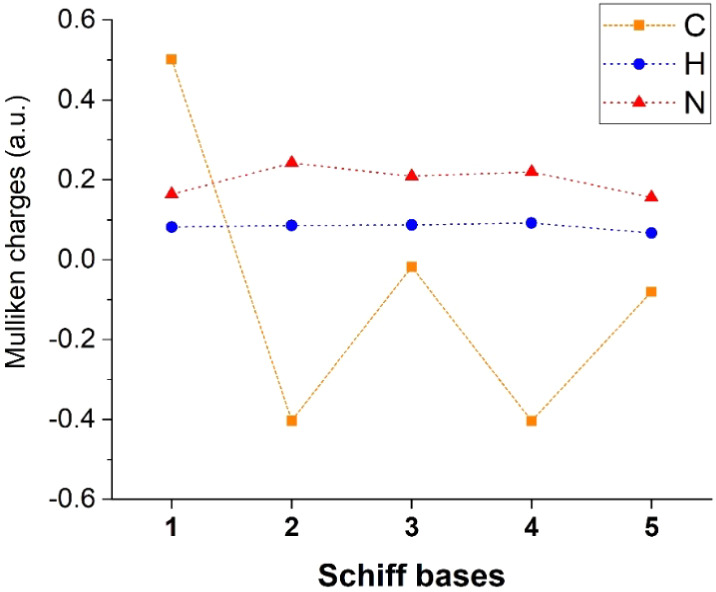
Mulliken charges of azomethine (HC=N) atoms C, H, and N of **1**–**5** showing pronounced substituent-induced variations at C and minimal changes at H and N sites.

The phenyl carbon directly bonded to the azomethine carbon (**C**_Ar′_) displays a pronounced positive charge in **2** (1.256) reflecting limited resonance donation by the methoxy group and a minimum for **5** (0.500) due to reduced π-conjugation in the absence of the methoxy substituent. In contrast, the carbon bonded to the azomethine nitrogen (**C**_Ar_) is consistently negatively charged, with the highest electron density in compound **1** (−0.707) and the lowest in **5** (−0.098) reflecting substituent-driven polarization along the C=N bond. In the methoxy-containing isomers **1**–**3**, the aryl carbon bonded to the methoxy group (**C**_Ar′_OCH_3_) carries a large negative charge (−1.068 (**1**), −0.346 (**2**), −0.622 (**3**)) confirming strong resonance donation into the aromatic π-system. The methoxy oxygen and carbon both remain negatively charged indicating pronounced polarization of the methoxy group. These features are not observed in **4** and **5**, consistent with the loss of resonance-assisted electron donation due to the lack of a methoxy substituent. The methyl-bearing aromatic carbons (**C**_Ar_Me) in compounds **1**–**4** display positive charge (0.312–0.475), while the methyl carbons (**C**H_3_) remain negatively charged (−0.498 to −0.570) consistent with a weak electron-donating inductive (+I) effect. The manifestation of this effect in **1**–**4,** and its absence in **5,** explains the graded attenuation of intramolecular charge transfer from the substituted to the unsubstituted system.

### 2.7 Frontier Molecular Orbitals (FMOs)

The DFT-computed FMOs are depicted in Figure S8. The energies of FMOs and the computed global molecular reactivity descriptors for Schiff bases **1**–**5** are summarized in Table S14 and their trends are depicted in Figure 12. The HOMO energy ordering (**1** > **3** > **2** > **4** > **5**) indicates that **1** is the strongest electron donor and **5** the weakest. The LUMO-based acceptor ranking (**1** > **5** > **4** > **2** > **3**) further highlights the dual character of **1** as both the strongest donor and acceptor. In contrast, compound **3**, despite having the most stabilized LUMO (−1.68 eV), exhibits relatively weak acceptor character. The pronounced asymmetric electronic polarization in **1**, evident from its MEP surface (Figure 2), is supported by its low HOMO–LUMO energy gap (ΔE = 1.70 eV) making the *ortho*-isomer **1** the most readily excitable π-conjugated system for potential nonlinear optical applications. Compound **1** exhibits a narrow ΔE (1.70 eV), whereas compounds **2**–**5** display substantially wider energy gaps (ΔE = 4.15–4.18 eV) with ∼2.5-fold increase. This distinct electronic behavior of **1** arises from the strong +M effect of the *ortho*-OCH_3_ group which enhances π-delocalization and facilitates intramolecular O···H interactions, improving orbital overlap and donor–acceptor coupling.^65^

**Figure 12:**
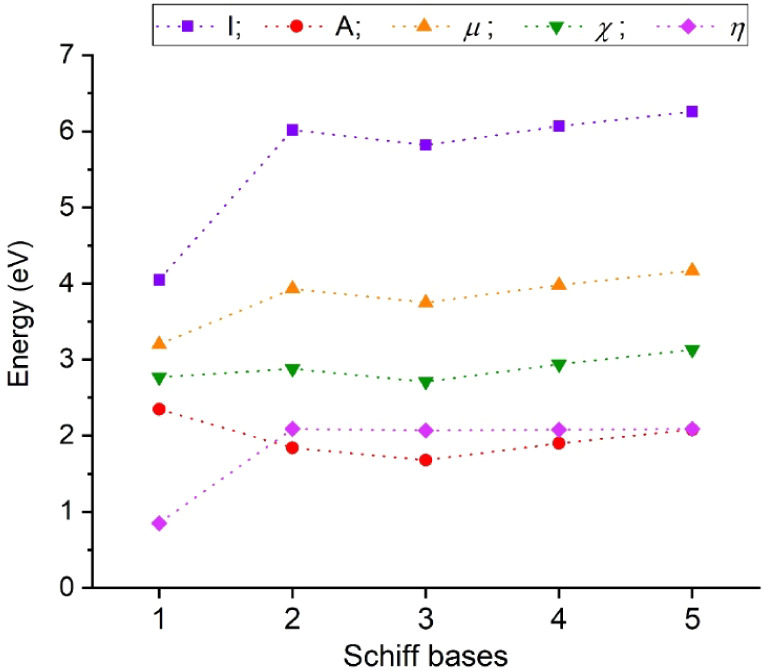
Trends in the DFT-computed ionization potential (I), electron affinity (A), chemical potential (μ), absolute electronegativity (χ), and absolute hardness (η) of Schiff bases **1**–**5**.

The global reactivity descriptors further corroborate these substituent-controlled trends in compounds **1**–**5**.^44,67–69^ Compound **1** shows the lowest ionization potential (I = 4.05 eV) and hardness (η = 0.85 eV) along with the highest electron affinity (A = 2.35 eV) and softness (σ = 1.17 eV^–1^), indicating greater chemical reactivity, polarizability, and electronic flexibility relative to compounds **2**–**5**. Consistent with its narrow ΔE and strong intramolecular charge transfer (**1** ≫ **3** ≈ **2** ≈ **4** ≈ **5**), compound **1** also exhibits the highest donor strength (**1** > **3** > **2** > **4** > **5**) and acceptor character (**1** > **5** > **4** > **2** > **3**), identifying it as the softest and most chemically reactive member of the series (**1** ≫ **2** ≈ **3** ≈ **4** ≈ **5**). In contrast, compound **5**, lacking both methoxy and methyl substituents, remains the least responsive, polarizable, and interactive, confirming that substituent identity and position govern the optical properties and reactivity of these systems. Thus, the global reactivity descriptors of compound **1** account for its superior electronic responsiveness.

### 2.8 Molecular Electronic Polarizability

The DFT-calculated dipole moment (μ), mean linear polarizability (α), and first nonlinear hyperpolarizability (β) of the Schiff bases **1**–**5** are summarized in Table S15 and their trend are depicted in Figure 13. The computed dipole moments of **1**–**5** reveal a strong dependence on both the nature and position of methoxy and methyl substituents. Among the methoxy-containing Schiff bases **1**–**3**, the dipole moments remain moderate (μ = 1.5–2.1 D), with the *meta*-isomer **2** exhibiting the highest value of μ = 2.1 D, followed by the *ortho*- (**1**, 1.6 D) and *para*- (**3**, 1.5 D) isomers. The absence of a methoxy group in **4** and both methoxy and methyl substituents in **5** results in the lowest μ values for **4** and **5** (∼1.3 D), highlighting the role of substituent-induced electronic asymmetry.

**Figure 13:**
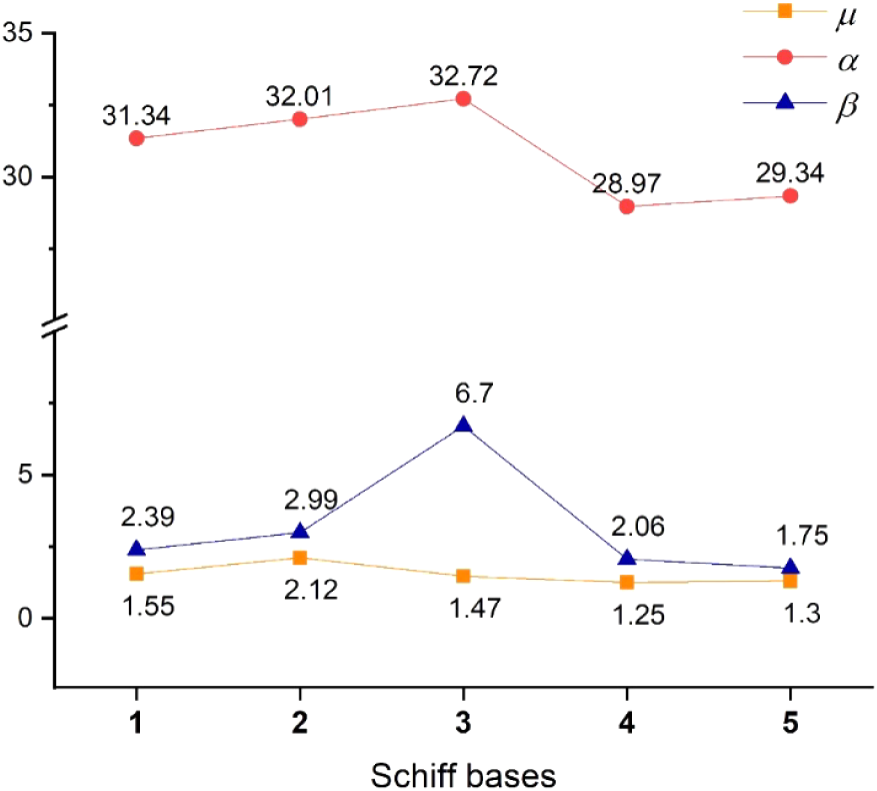
Trends in DFT-computed dipole moment (μ), mean linear polarizability (α), and first nonlinear hyperpolarizability (β) of Schiff bases **1**–**5**.

The mean linear polarizability (α) of **1**–**5** reflects the combined effects of π-conjugation, substituent electron donor ability, and molecular size.^70–72^ Among the Schiff bases **1**–**5**, α increases systematically from the unsubstituted systems **4** (29.0 × 10^–24^ esu) and **5** (29.3 × 10^–24^ esu) to the methoxy-substituted derivatives **1**–**3** reaching a maximum for the *para*-methoxy compound **3** (32.7 × 10^–24^ esu). This trend underscores the effectiveness of *para*-substitution in enhancing resonance-assisted π-delocalization and asymmetric polarization. The *ortho*- and *meta*-methoxy analogues **1** and **2** show slightly lower α values reflecting reduced conjugation efficiency. These results indicate that α is predominantly controlled by extended conjugation favored by the methoxy group in **1**–**3** and substituent-induced electronic polarization.

The first nonlinear hyperpolarizability coefficient (β) indicates the most pronounced sensitivity to substituent effects. Within the methoxy-substituted compounds **1**–**3**, the β value increases from the *ortho*- (**1**, 2.4 × 10^–30^ esu) to the *meta*-isomer (**2**, 3.0 × 10^–30^ esu) with a substantial increase for the *para*-derivative **3** (6.7 × 10^–30^ esu), confirming that the *para*-substitution optimally aligns donor orbitals with the π-conjugated framework. The absence of the methoxy group in **4** and **5** leads to significantly reduced β values (≤2.1 × 10^–30^ esu), highlighting the critical role of electron donation in NLO enhancement. Substituent effects in modulating the DFT-calculated molecular electronic polarizability parameters are further illustrated by the related Schiff bases **S1** and **S2** (Figure S9) with nitro and chloro substituents instead of the methoxy group in **3**. The electron-withdrawing nitro and chloro substituents enhance the electronic response in the Schiff bases **S1** and **S2** relative to the *p*-methoxy analogue **3**. The nitro derivative exhibits the most pronounced effect (μ = 6.3 D; α = 34.0 × 10^–24^ esu; β = 11.2 × 10^–30^ esu) consistent with strong ground-state charge separation and an efficient push–pull (D−π–A) framework, whereas the chloro analogue shows a moderate enhancement (μ = 2.2 D; α = 32.0 × 10^–24^ esu; β = 1.9 × 10^–30^ esu) attributable to its −I/+M character.^33^

## 3 Conclusions

A combined experimental and DFT/TD-DFT investigation of methoxy-substituted *N*-benzylideneaniline Schiff bases **1**–**3** in comparison with unsubstituted analogues **4** and **5** demonstrates that substituent identity and positional orientation strongly modulate the electronic behavior of the azomethine framework. FT-IR analysis identifies the *ν*(C=N) vibration as the most responsive structural probe with the *ortho*-methoxy derivative **1** showing a slight red-shift (1632 cm^–1^), while the *para*-substituted analogue **3** exhibits a relatively blue-shifted (1659 cm^–1^) more intense band due to enhanced resonance donation. ^1^H NMR spectra reveal that compound **1** experiences the strongest electronic perturbation displaying the most downfield azomethine proton signal (*δ* ≈ 8.9 ppm) compared to **2** and **3**, whereas **4** and **5** remain comparatively unchanged. ^13^C NMR spectra also show the most pronounced shielding of the azomethine carbon for **1** (δ ≈ 156 ppm) relative to the other derivatives confirming stronger +R effect and anisotropic influence of the *ortho*-methoxy substituent. Electronic absorption spectral study reveals strong *π* → π* transitions for methoxy derivatives **1**–**3** governed by ICT following the order: **3** > **1** ≫ **2**, whereas solvatochromic sensitivity is the highest for **1**, particularly in polar aprotic solvents.

Despite these electronic differences, optimized structures show nearly identical C=N bond lengths (1.276–1.278 Å) indicating that substituent effects primarily alter electron distribution rather than molecular geometry. MEP and Mulliken charge analyses confirm enhanced electronic polarization in methoxy derivatives, while frontier orbital calculations show that compound **1** possesses the smallest HOMO–LUMO energy gap (ΔE ≈ 1.70 eV) and the strongest ICT response (**1** ≫ **3** ≈ **2** ≈ **4** ≈ **5**). Nonlinear optical calculations further indicate that *para*-methoxy substitution maximizes optical nonlinearity with the highest β value of 6.7 × 10^–30^ esu for **3**. Overall, the study establishes a clear structure–electronic property relationship in regioisomeric Schiff bases, identifying **1** as the most electronically responsive system and **3** as the most efficient NLO-active derivative. The ICT behavior, solvatochromic sensitivity, and enhanced nonlinear optical responses highlight the potential of these methoxy-engineered azomethine systems for optoelectronic and nonlinear optical applications, thereby demonstrating the effectiveness of a combined experimental and computational design strategy.

## 4 Experimental Section

### 4.1 Chemicals and Reagents

4-Methylaniline (*p*-toluidine) (CAS No. 106-49-0), 2-, 3-, and 4-Methoxybenzaldehydes (*o*-/*m*-/*p*-Anisaldehyde) (CAS Nos. 135-02-4 (*ortho-*), 591-31-1 (*meta-*), and 123-11-5 (*para-*)), Benzaldehyde (CAS No. 100-52-7), and Aniline (CAS No. 62-53-3) were procured from Sigma-Aldrich and used as received. All solvents employed were of analytical grade or the highest purity available and were used without further purification.

### 4.2 Spectral Studies

Infrared spectra were recorded as KBr pellets on a Bruker Tensor II FT-IR spectrometer in the range 4000–400 cm^–1^. The ^1^H and ^13^C NMR spectra were recorded in CDCl_3_ on a Bruker AVNEO 500 MHz NMR spectrometer at 298 K. The electronic absorption spectra were recorded on an Agilent Cary 8454 UV–visible spectrophotometer operated under ChemStation Version B.05.02 software. The solvatochromic measurements were carried out in solution over the range 190–1100 nm at 298 K using a matched pair of Teflon-stoppered quartz cuvettes with a path length of 1 cm. A stock solution of the title compound in acetonitrile (9.87 × 10^–5^ M) was used to prepare working solutions of 2.50 × 10^–5^ M. These were obtained by transferring an appropriate aliquot of the stock solution into a 10 mL volumetric flask, evaporating the solvent to dryness, and reconstituting to volume with the respective solvents. The solutions were thoroughly mixed to ensure homogeneity. Baseline correction was performed using the corresponding pure solvent prior to spectral acquisition.

### 4.3 DFT Study

Density functional theory (DFT) calculations were performed using the Gaussian 16 program (Revision A.03, x86–64 AVX2-enabled/Linux) along with the GaussView 6.0.16 graphical interface.^73^ Geometry optimizations and electronic structure calculations were carried out employing the hybrid functional B3LYP in conjunction with the 6-311++G­(d,p) triple-ζ Pople basis set. This basis set includes diffuse functions (“++”) on all atoms and polarization functions (d,p), providing an improved description of the electron distribution.^74–77^ The ^1^H and ^13^C NMR chemical shifts of compounds **1**–**5** were calculated using the self-consistent field gauge-independent atomic orbital (SCF-GIAO) method, with tetramethylsilane (TMS) as the reference. The chemical shifts of TMS were computed at the B3LYP/6-311+G­(2d,p) level.^78^ Time-dependent DFT (TD-DFT) calculations were employed to simulate the electronic absorption spectra of the studied molecules in the gas phase as well as in various solvent environments.^24,39,51,52^

### 4.4 General Procedure for the Preparation of Schiff Bases **1**–**5**

Schiff bases **1**–**3** were synthesized by condensing 2-, 3-, or 4-methoxybenzaldehyde with 4-methylaniline in a 1:1 molar ratio in dry dichloromethane, while Schiff bases **4** and **5** were synthesized by the condensation of benzaldehyde with 4-methylaniline and aniline, respectively. Anhydrous sodium sulfate was employed as a dehydrating agent to remove the water formed during the reaction. In a typical procedure,^54,60,61,79^ *p*-toluidine (0.5360 g, 5 mmol) was dissolved in dry dichloromethane (20 mL) and a solution of *o*-, *m*-, or *p*-anisaldehyde (0.6810 g, 5 mmol) in dry dichloromethane (20 mL) was added dropwise under stirring to afford compounds **1**–**3**. Compounds **4** and **5** were prepared analogously using benzaldehyde (5 mmol) with *p*-toluidine or aniline (5 mmol), respectively. The reaction mixtures were refluxed for 3 h in the presence of anhydrous sodium sulfate (0.50 g), cooled to room temperature, filtered, and concentrated under reduced pressure. The crude products were obtained in high yields (>90%) and purified by recrystallization from a hot hexane–ethanol (1:1, v/v) mixture.

## Supplementary Material



## Data Availability

The spectroscopic and DFT/TD-DFT computed data for this study are available in the published article and its Supporting Information.

## Notes

FT-IR and NMR spectra, images of FMOs, DFT-calculated structural parameters, Mulliken charges, dihedral angles, and experimental and calculated spectral data of **1**–**5** (PDF)
