# Supplementary material for: Experimental and DFT/TD-DFT Insights into Spectroscopic, Solvatochromic, and Nonlinear Optical Properties of Methoxy-Substituted N‑Benzylideneaniline Schiff Base Regioisomers
Source: ACS Omega. 2026 Jul 8;11(28):42208–21. doi: 10.1021/acsomega.6c02717 (PMC13393185; doi:10.1021/acsomega.6c02717)
Supplement: Supplementary file 1 [file ao6c02717_si_001.pdf]

Supplementary Information

Experimental and DFT/TD-DFT Insights into Spectroscopic, Solvatochromic, and Nonlinear Optical Properties of Methoxy-Substituted *N*-Benzylideneaniline Schiff Base Regioisomers

Wilson Bosco Paul Michael,[†] Hemamalini Abraham,[‡] Selvam Amudhan Senthan,[§] Carlson Alexander,[⊥*] and A. Irudaya Jothi[‡*]

[†] *Department of Chemistry, Sacred Heart College, Tirupattur 635601, Tamil Nadu, India.*

[‡] *Department of Chemistry, St. Joseph's College, Tiruchirappalli 620002 (Affiliated to Bharathidasan University, Tiruchirappalli 620024), Tamil Nadu, India.*

[§] *NAWaL Analytical Labs India Pvt. Ltd., Electrical & Electronics Industrial Estate, Hosur 635109, Tamil Nadu, India.*

[⊥] *Department of Chemistry, Hong Kong Baptist University, Ho Sing Hang Campus, 224 Waterloo Road, Kowloon Tong, Hong Kong SAR 999077, China.*

Correspondence to: carlsonalex@hkbu.edu.hk, irudayajyothi@gmail.com

Content

	Page
Fig. S1. Representative resonance contributors for compounds **1**–**3**	S2
Fig. S2. FT-IR spectra of **1**–**3**	S3
Fig. S3. FT-IR spectra of **4** and **5**	S4
Fig. S4. ^{1}H and ^{13}C spectra of **1**	S5
Fig. S5. ^{1}H and ^{13}C spectra of **2**	S6
Fig. S6. ^{1}H and ^{13}C spectra of **3**	S7
Fig. S7. ^{1}H and ^{13}C spectra of **4**	S8
Fig. S8. FMOs of **1**–**5** computed at the B3LYP/6-311++G(d,p)) level	S9
Fig. S9. Structures of the nitro- and chloro analogues **S1** and **S2** of Schiff base **3**	S10
Table S1. DFT calculated bond lengths, angles, and Mulliken charges for **1**	S11
Table S2. DFT calculated bond lengths, angles, and Mulliken charges for **2**	S12
Table S3. DFT calculated bond lengths, angles, and Mulliken charges for **3**	S13
Table S4. DFT calculated bond lengths, angles, and Mulliken charges for **4**	S14
Table S5. DFT calculated bond lengths, angles, and Mulliken charges for **5**	S15
Table S6. DFT calculated dihedral angles for **1**–**5**	S16
Table S7. DFT-calculated dihedral angles for **4** and **5**	S18
Table S8. Characteristic FT-IR absorption bands (cm^{-1}) of Schiff bases **1**–**5**	S20
Table S9. Experimental and DFT-calculated ^{1}H chemical shifts for **1**–**5**	S21
Table S10. Experimental and DFT-calculated ^{13}C NMR chemical shifts for **1**–**5**	S22
Table S11. Electronic absorption spectral data for **1**–**5** in different solvents	S23
Table S12. TD-DFT-simulated absorption spectral data for **1**–**5** in different solvents	S24
Table S13. DFT calculated Mulliken atomic charges for key atoms of Schiff bases **1**–**5**	S25
Table S14. DFT-calculated FMO energies and global reactivity descriptors of **1**–**5**	S26
Table S15. DFT-calculated dipole moment (μ), linear polarizability (α), and first hyperpolarizability (β) of **1**–**5**	S27

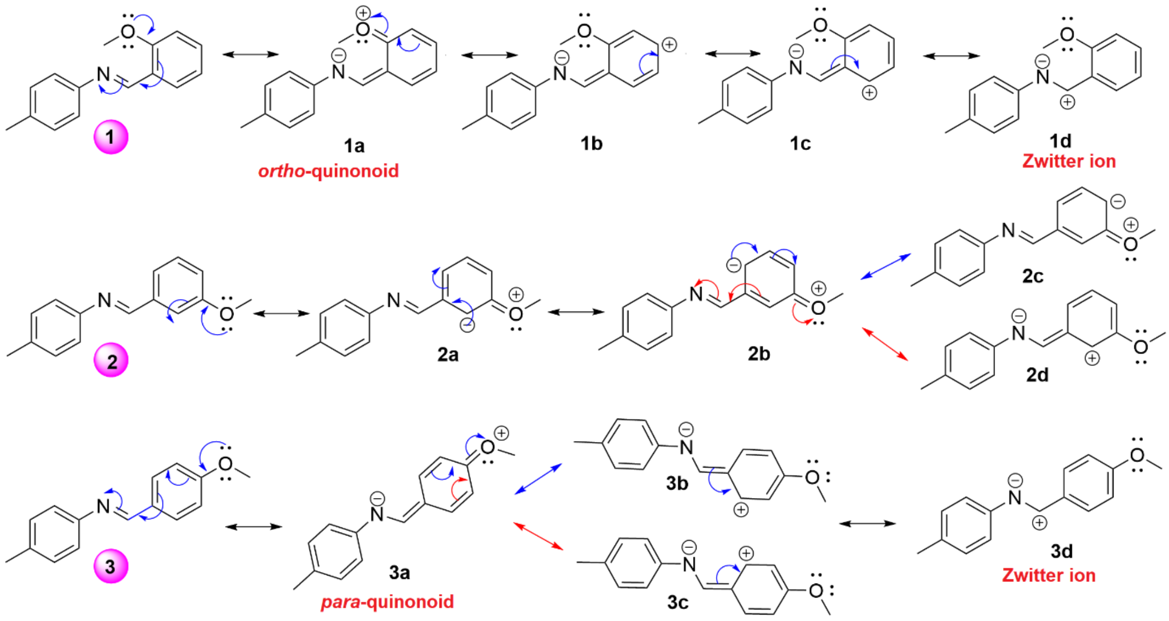

Fig. S1. Representative resonance contributors for compounds **1**–**3** illustrating the delocalization of π-electron density across the aromatic rings and the imine functionality. These canonical forms rationalize the observed structural features, including partial double-bond character at the C=N linkage and variations in adjacent bond lengths within the conjugated backbone.

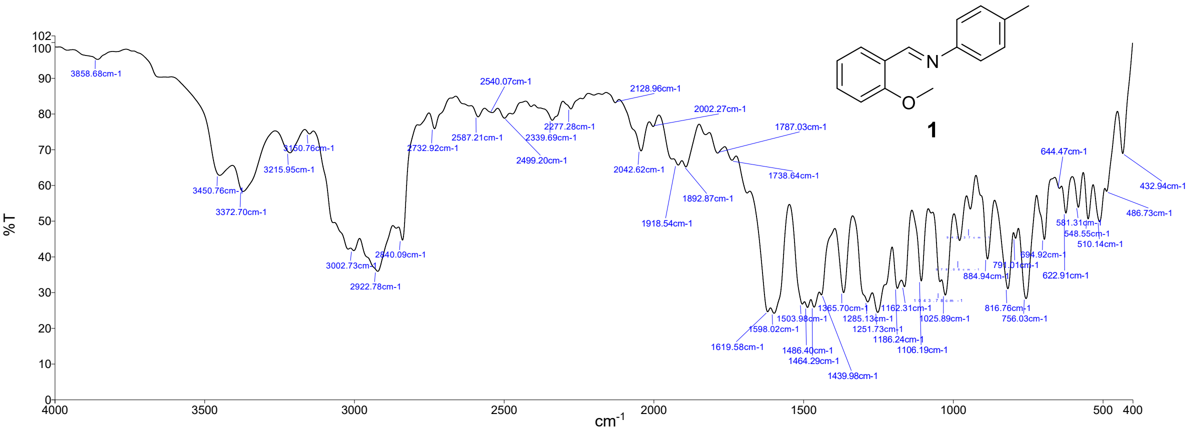

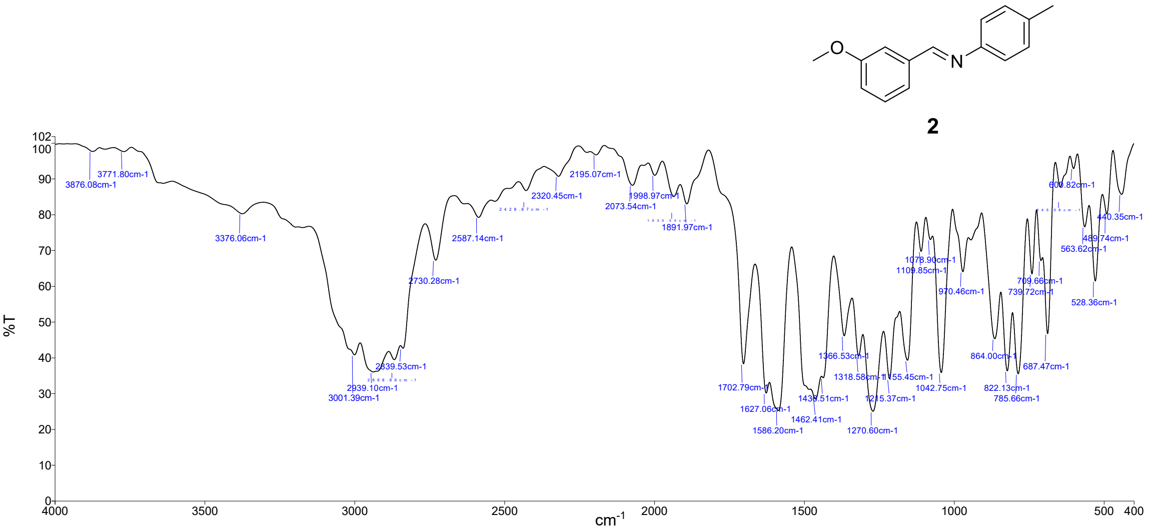

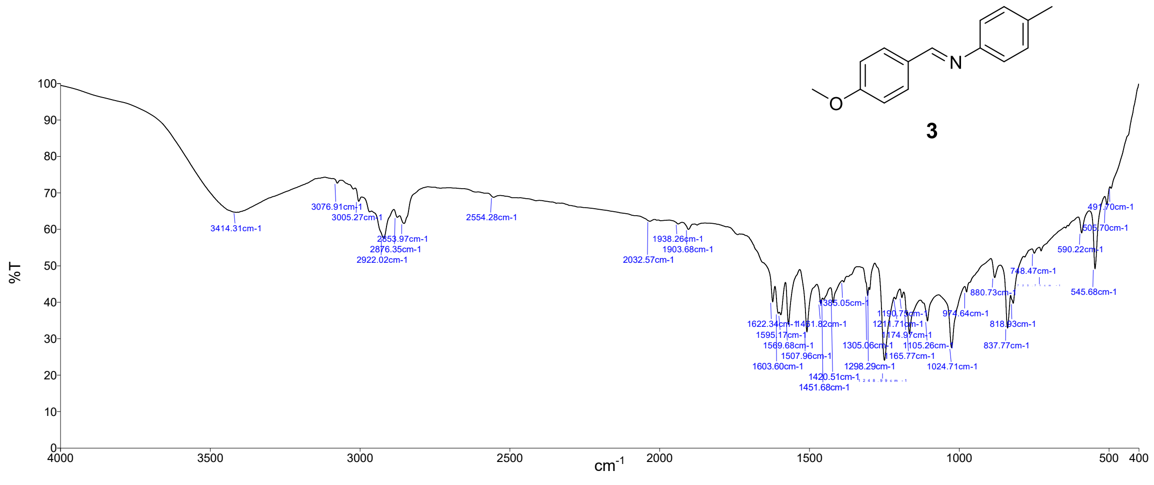

Fig. S2. FT-IR spectra of **1**–**3**.

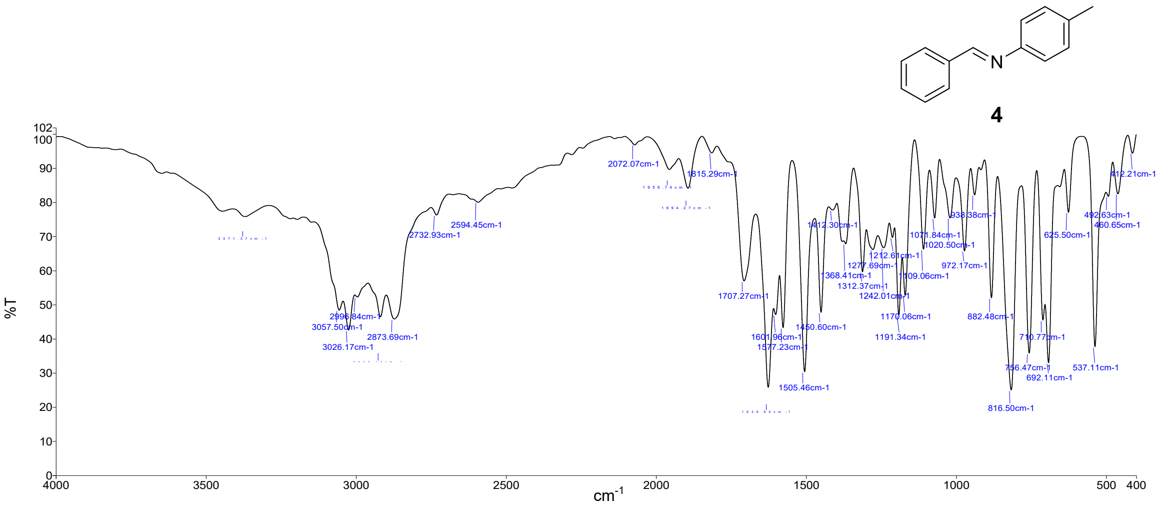

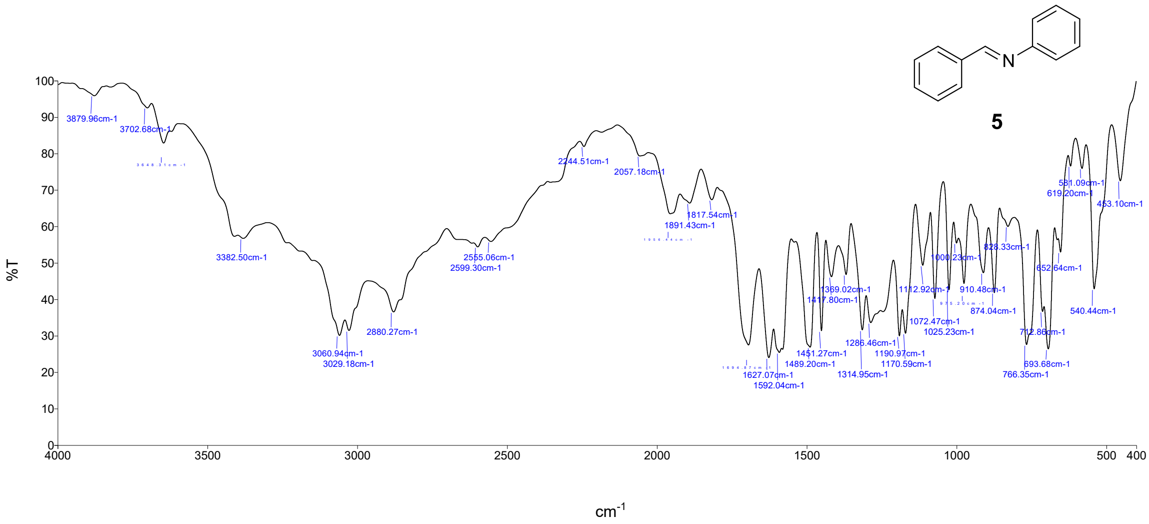

Fig. S3. FT-IR spectra of **4** and **5**.

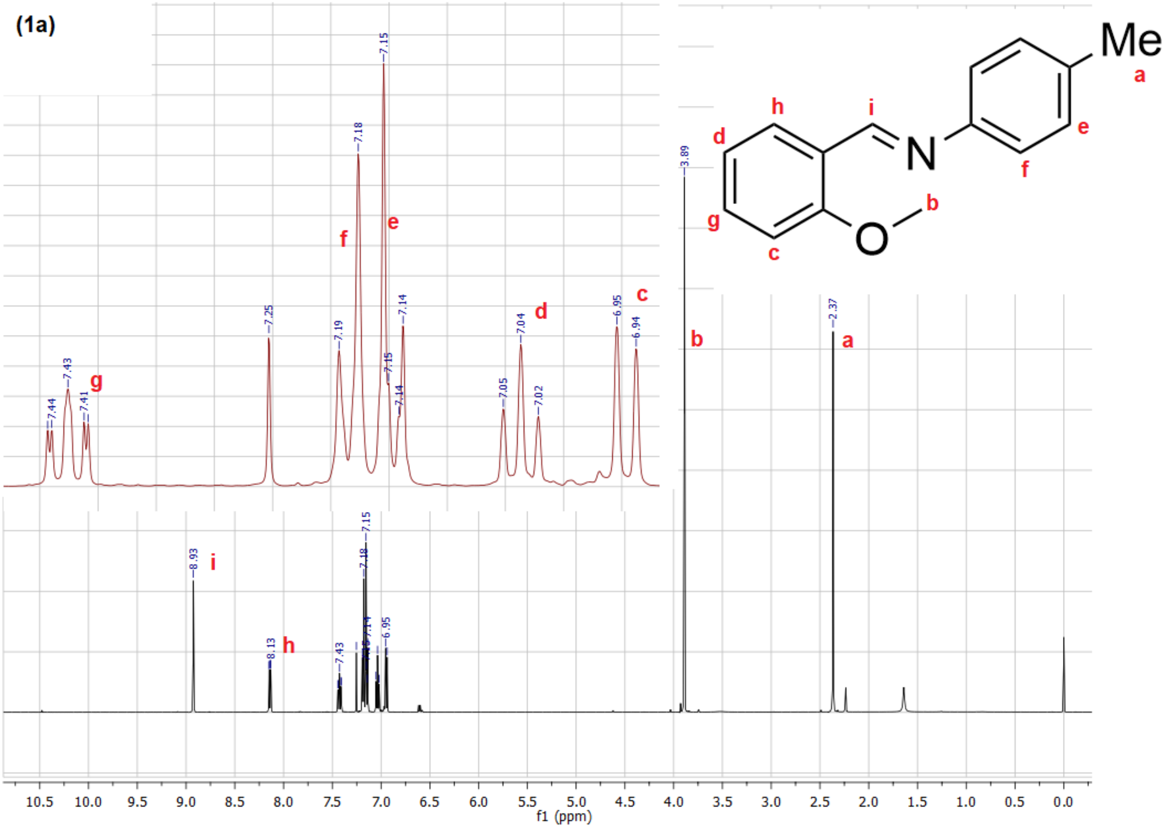

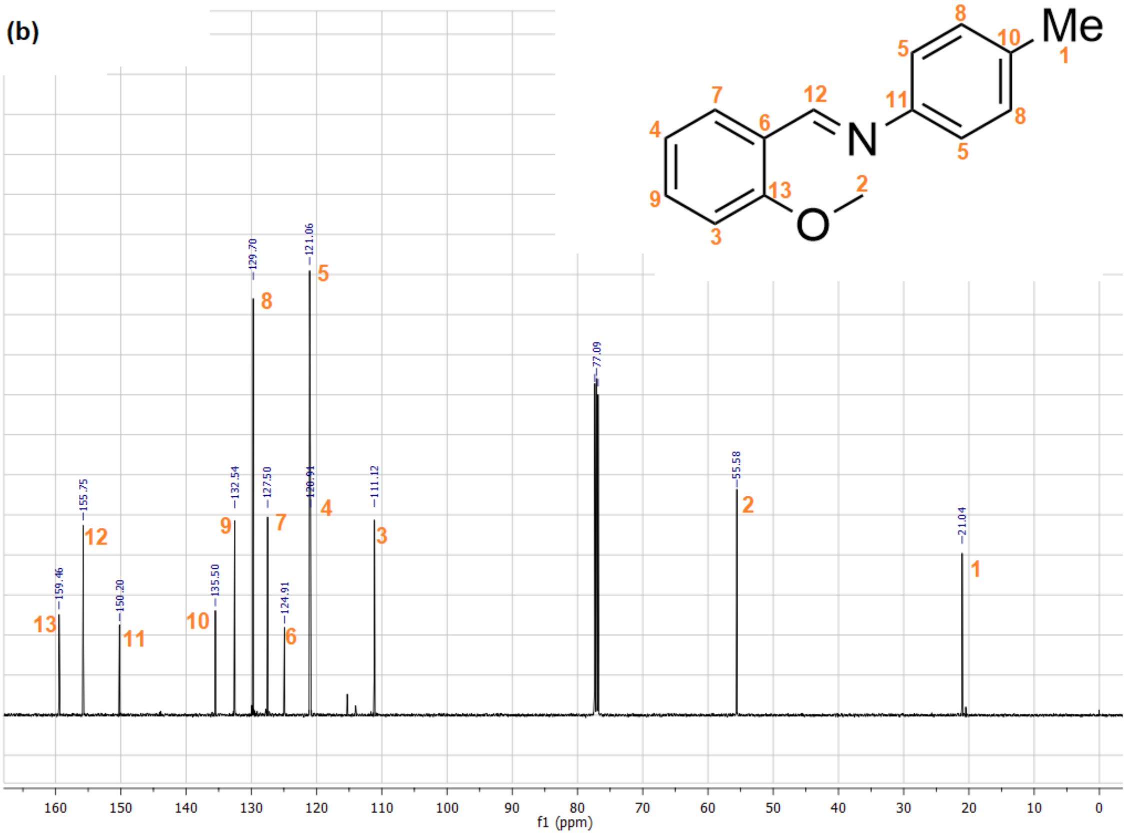

Fig. S4. (a) ^{1}H and (b) ^{13}C NMR spectra of **1**.

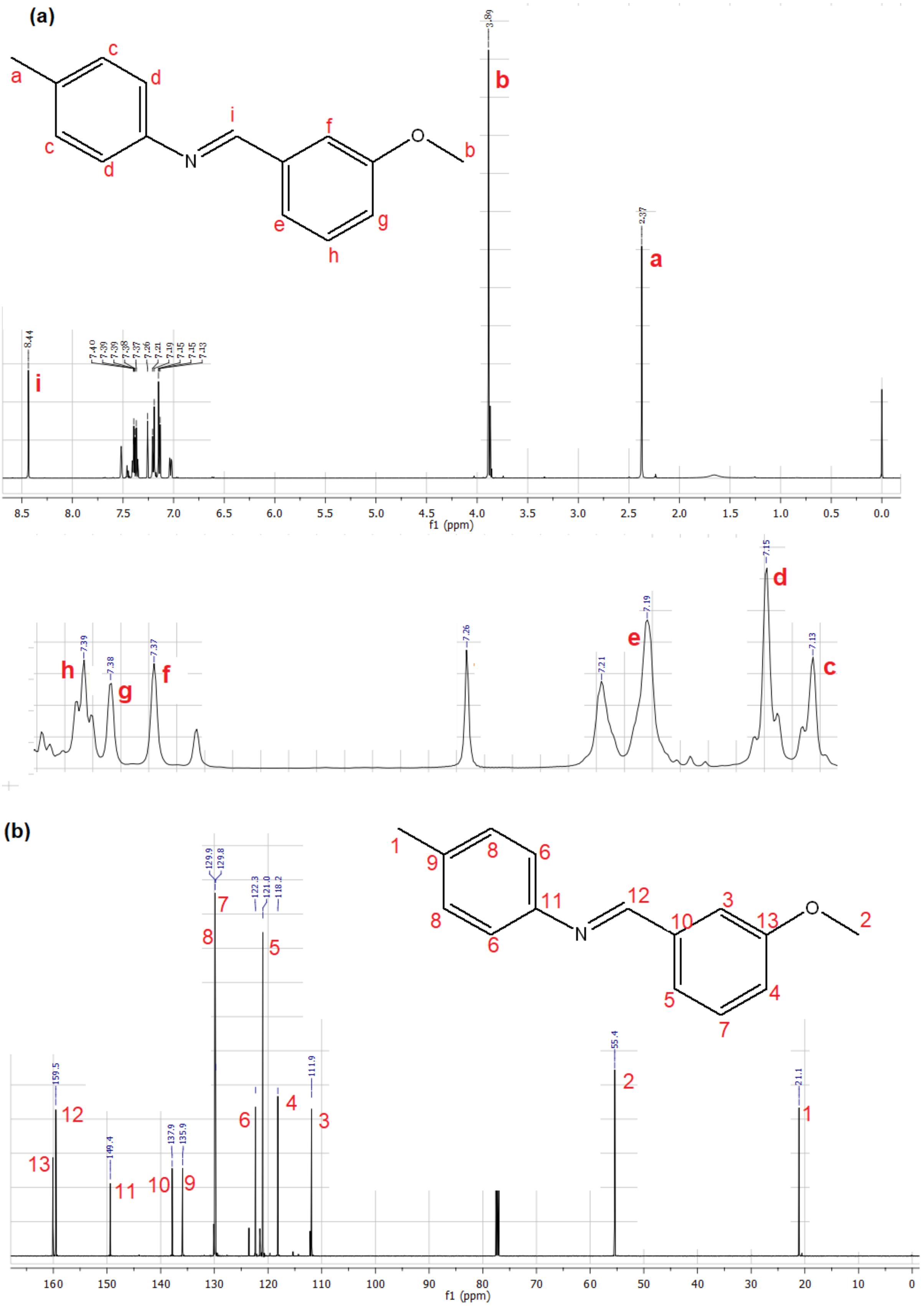

Fig. S5. (a) ^{1}H and (b) ^{13}C NMR spectra of **2**.

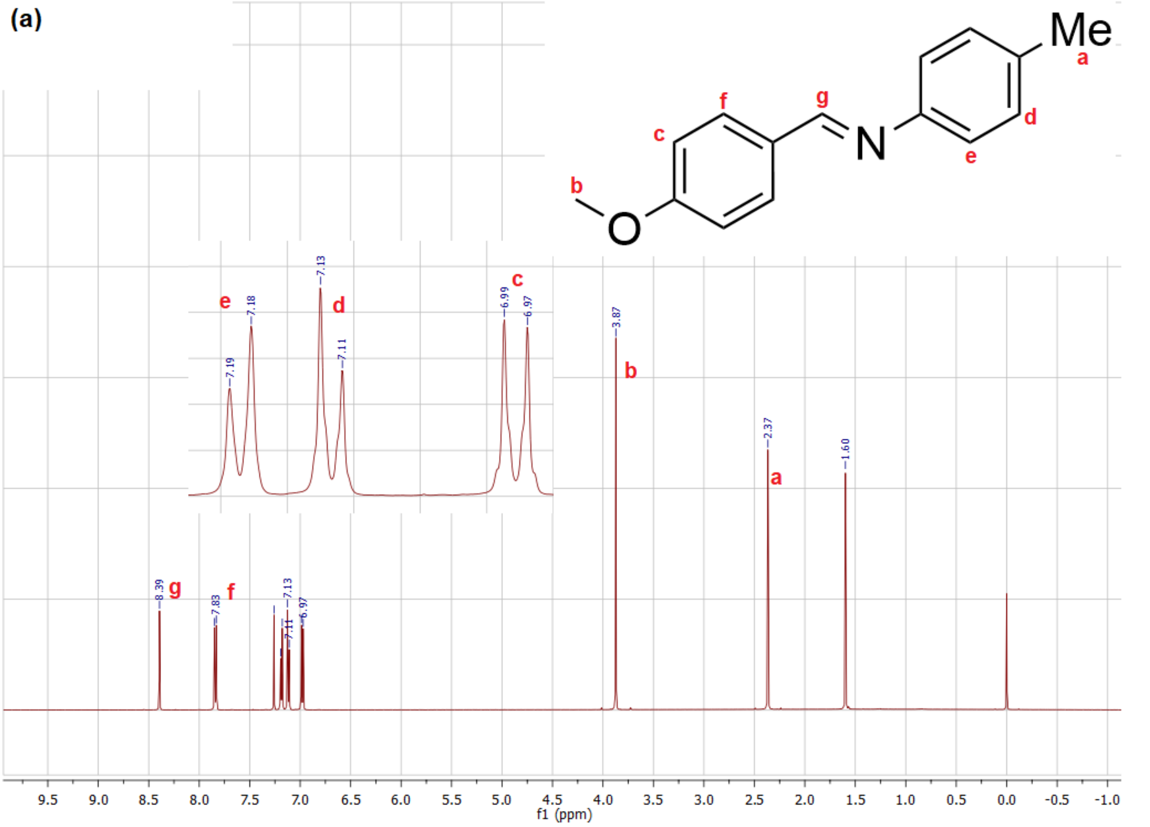

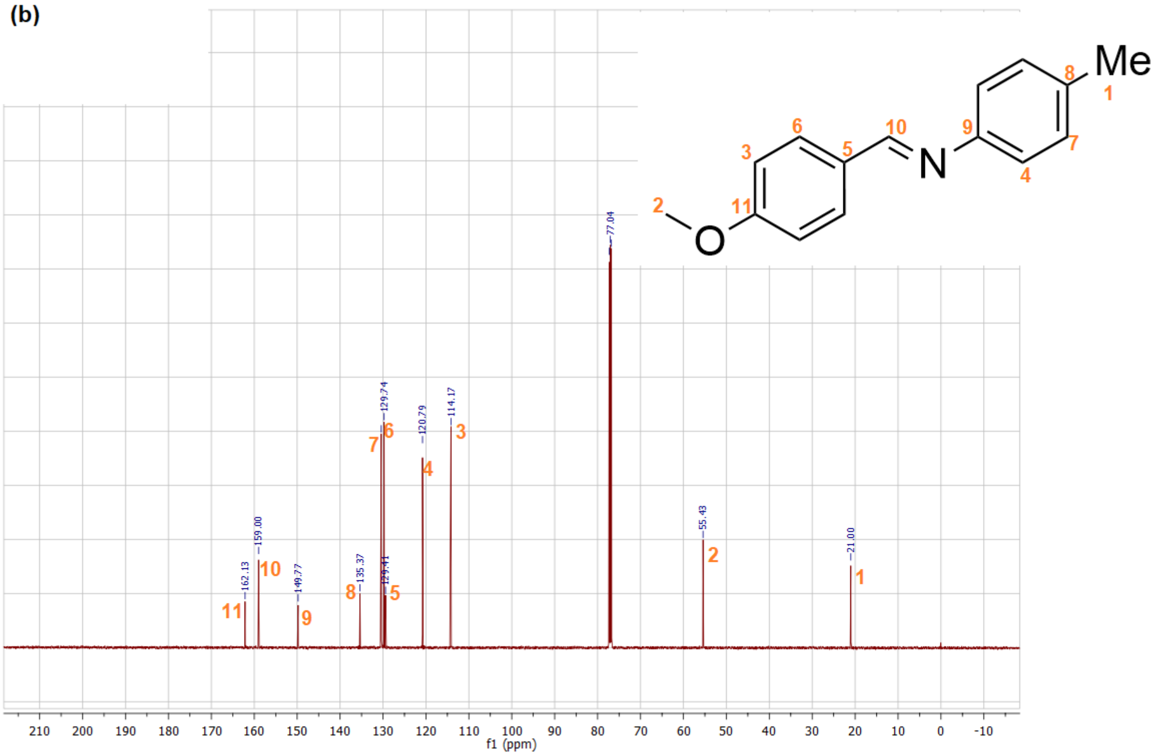

Fig. S6. (a) ^{1}H and (b) ^{13}C NMR spectra of **3**.

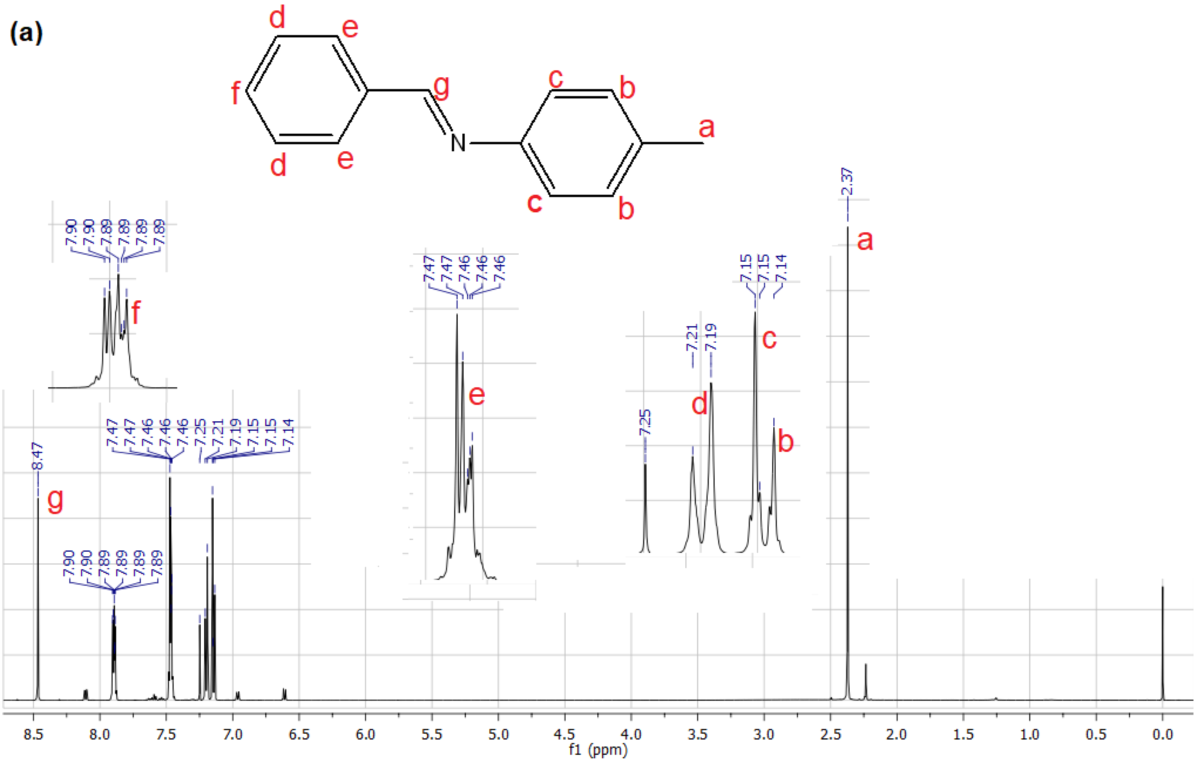

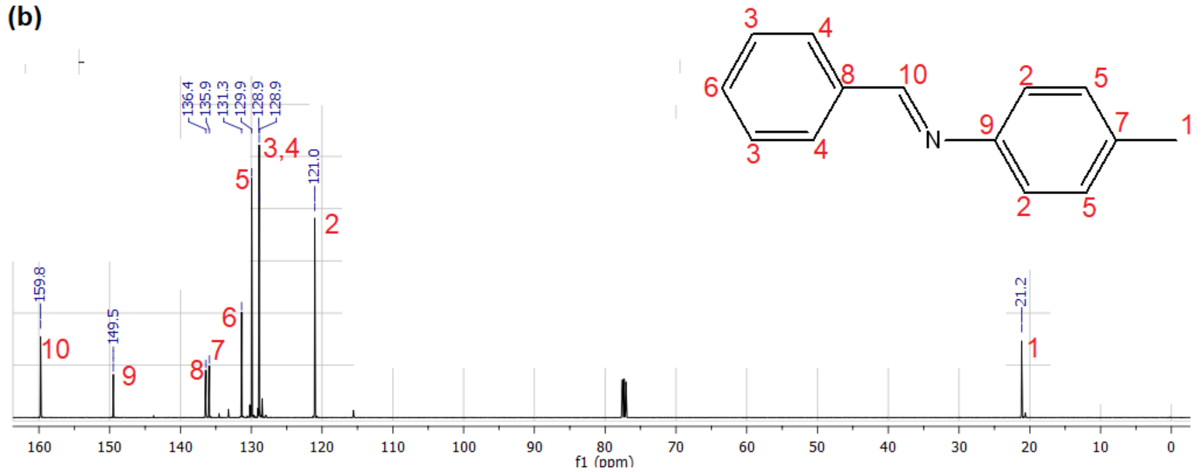

Fig. S7. (a) ^{1}H and (b) ^{13}C NMR spectra of **4**.

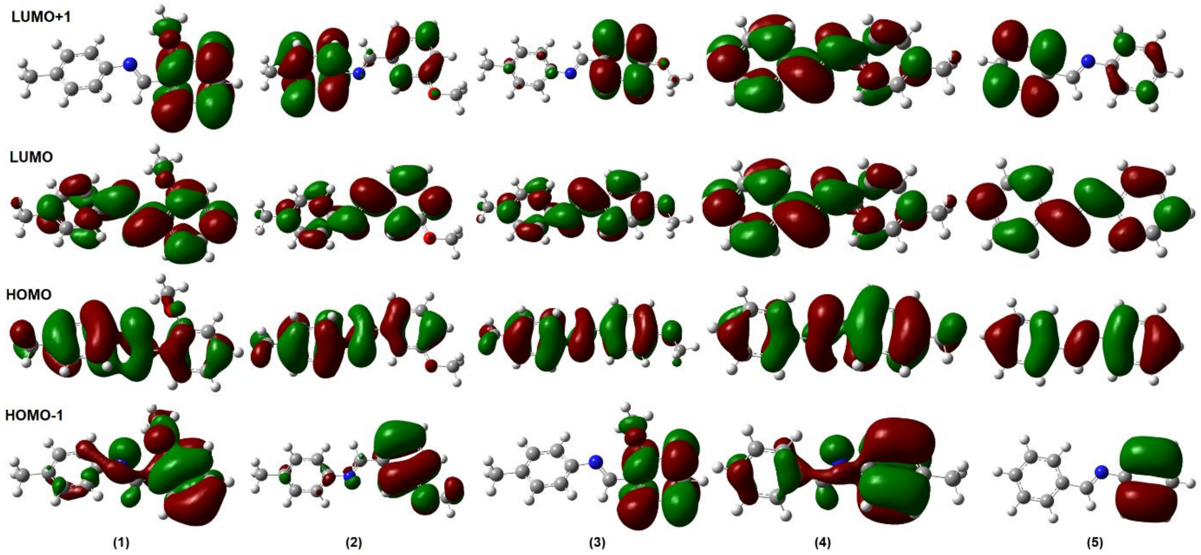

Fig. S8. Frontier molecular orbitals of **1**–**5** computed at the B3LYP/6-311++G(d,p)) level.

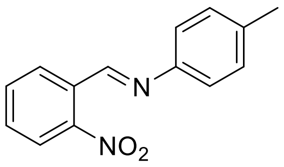

S1

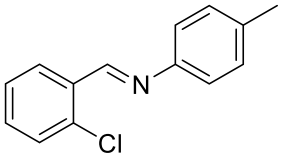

S2

Fig. S9. Structures of the nitro- and chloro analogues **S1** and **S2** of **3**.

Table S1. DFT calculated bond lengths, bond angles, and Mulliken charges for **1**

Bond Length	Definition	Value	Bond Angle	Definition	Value	Atom	Mulliken charge
R1	R(1,2)	1.4017	A1	A(2,1,6)	117.72	1C	0.4117
R2	R(1,6)	1.3973	A2	A(2,1,23)	120.88	2C	-0.3015
R3	R(1,23)	1.5095	A3	A(6,1,23)	121.37	3C	0.5633
R4	R(2,3)	1.3884	A4	A(1,2,3)	121.36	4C	-0.7070
R5	R(2,7)	1.0858	A5	A(1,2,7)	119.42	5C	-0.4485
R6	R(3,4)	1.4027	A6	A(3,2,7)	119.22	6C	-0.2496
R7	R(3,8)	1.0839	A7	A(2,3,4)	120.60	7H	0.1793
R8	R(4,5)	1.4019	A8	A(2,3,8)	120.70	8H	0.1412
R9	R(4,11)	1.4064	A9	A(4,3,8)	118.69	9H	0.1631
R10	R(5,6)	1.3931	A10	A(3,4,5)	118.39	10H	0.1508
R11	R(5,9)	1.0842	A11	A(3,4,11)	118.32	11N	0.1635
R12	R(6,10)	1.0856	A12	A(5,4,11)	123.23	12C	0.5016
R13	R(11,12)	1.2769	A13	A(4,5,6)	120.43	13H	0.0816
R14	R(12,13)	1.0995	A14	A(4,5,9)	119.72	14C	1.0265
R15	R(12,14)	1.4681	A15	A(6,5,9)	119.81	15C	-0.5753
R16	R(14,15)	1.4068	A16	A(1,6,5)	121.45	16C	-1.0679
R17	R(14,16)	1.4148	A17	A(1,6,10)	119.42	17C	-0.4784
R18	R(15,17)	1.3883	A18	A(5,6,10)	119.13	18H	0.1574
R19	R(15,18)	1.0854	A19	A(4,11,12)	119.49	19C	-0.0251
R20	R(16,19)	1.3956	A20	A(11,12,13)	120.59	20C	-0.2380
R21	R(16,28)	1.3682	A21	A(11,12,14)	125.74	21H	0.1619
R22	R(17,20)	1.3940	A22	A(13,12,14)	113.65	22H	0.1946
R23	R(17,21)	1.0836	A23	A(12,14,15)	116.59	23C	-0.4983
R24	R(19,20)	1.3918	A24	A(12,14,16)	125.55	24H	0.1667
R25	R(19,22)	1.0839	A25	A(15,14,16)	117.82	25H	0.1422
R26	R(20,27)	1.0843	A26	A(14,15,17)	122.12	26H	0.1463
R27	R(23,24)	1.0955	A27	A(14,15,18)	118.33	27H	0.1672
R28	R(23,25)	1.0936	A28	A(17,15,18)	119.55	28O	-0.0799
R29	R(23,26)	1.0921	A29	A(14,16,19)	119.83	29C	-0.2840
R30	R(28,29)	1.4325	A30	A(14,16,28)	122.32	30H	0.1232
R31	R(29,30)	1.0960	A31	A(19,16,28)	117.68	31H	0.1491
R32	R(29,31)	1.0914	A32	A(15,17,20)	119.28	32H	0.1625
R33	R(29,32)	1.0893	A33	A(15,17,21)	120.19		
			A34	A(20,17,21)	120.53		
			A35	A(16,19,20)	121.10		
			A36	A(16,19,22)	117.93		
			A37	A(20,19,22)	120.96		
			A38	A(17,20,19)	119.84		
			A39	A(17,20,27)	120.34		
			A40	A(19,20,27)	119.81		
			A41	A(1,23,24)	111.22		
			A42	A(1,23,25)	111.36		
			A43	A(1,23,26)	111.36		
			A44	A(24,23,25)	107.21		
			A45	A(24,23,26)	107.54		
			A46	A(25,23,26)	107.96		
			A47	A(16,28,29)	116.16		
			A48	A(28,29,30)	110.49		
			A49	A(28,29,31)	110.66		
			A50	A(28,29,32)	105.99		
			A51	A(30,29,31)	110.63		
			A52	A(30,29,32)	109.55		
			A53	A(31,29,32)	109.40		

Table S2. DFT calculated bond lengths, bond angles, and Mulliken charges for **2**

Bond Length	Definition	Values	Bond angle	Definition	Values	Atom	Mulliken charge
R1	R(1,2)	1.4020	A1	A(2,1,6)	117.73	1C	0.3118
R2	R(1,6)	1.3972	A2	A(2,1,24)	120.81	2C	0.0479
R3	R(1,24)	1.5095	A3	A(6,1,24)	121.46	3C	0.1200
R4	R(2,3)	1.3881	A4	A(1,2,3)	121.35	4C	-0.3014
R5	R(2,7)	1.0858	A5	A(1,2,7)	119.43	5C	-0.2437
R6	R(3,4)	1.4030	A6	A(3,2,7)	119.22	6C	-0.4696
R7	R(3,8)	1.0838	A7	A(2,3,4)	120.60	7H	0.1779
R8	R(4,5)	1.4016	A8	A(2,3,8)	120.88	8H	0.1631
R9	R(4,11)	1.4057	A9	A(4,3,8)	118.52	9H	0.1600
R10	R(5,6)	1.3934	A10	A(3,4,5)	118.41	10H	0.1413
R11	R(5,9)	1.0842	A11	A(3,4,11)	118.02	11N	0.2416
R12	R(6,10)	1.0855	A12	A(5,4,11)	123.50	12C	-0.4031
R13	R(11,12)	1.2760	A13	A(4,5,6)	120.41	13H	0.0859
R14	R(12,13)	1.0987	A14	A(4,5,9)	119.81	14C	1.2662
R15	R(12,14)	1.4698	A15	A(6,5,9)	119.74	15C	-0.7591
R16	R(14,15)	1.4045	A16	A(1,6,5)	121.45	16C	-0.7086
R17	R(14,16)	1.3962	A17	A(1,6,10)	119.41	17C	-0.3479
R18	R(15,17)	1.3885	A18	A(5,6,10)	119.13	18H	0.1433
R19	R(15,18)	1.0845	A19	A(4,11,12)	120.35	19C	-0.3461
R20	R(16,19)	1.3949	A20	A(11,12,13)	121.58	20H	0.2285
R21	R(16,20)	1.0824	A21	A(11,12,14)	123.05	21C	0.2449
R22	R(17,21)	1.3957	A22	A(13,12,14)	115.36	22H	0.1740
R23	R(17,22)	1.0842	A23	A(12,14,15)	119.04	23O	-0.1590
R24	R(19,21)	1.4012	A24	A(12,14,16)	121.29	24C	-0.5704
R25	R(19,23)	1.3647	A25	A(15,14,16)	119.67	25H	0.1593
R26	R(21,32)	1.0816	A26	A(14,15,17)	119.78	26H	0.1499
R27	R(23,28)	1.4210	A27	A(14,15,18)	119.87	27H	0.1489
R28	R(24,25)	1.0950	A28	A(17,15,18)	120.35	28C	-0.3084
R29	R(24,26)	1.0943	A29	A(14,16,19)	120.29	29H	0.1508
R30	R(24,27)	1.0919	A30	A(14,16,20)	119.99	30H	0.1516
R31	R(28,29)	1.0955	A31	A(19,16,20)	119.72	31H	0.1770
R32	R(28,30)	1.0955	A32	A(15,17,21)	120.78	32H	0.1736
R33	R(28,31)	1.0887	A33	A(15,17,22)	120.04		
			A34	A(21,17,22)	119.18		
			A35	A(16,19,21)	120.03		
			A36	A(16,19,23)	115.76		
			A37	A(21,19,23)	124.21		
			A38	A(17,21,19)	119.44		
			A39	A(17,21,32)	119.49		
			A40	A(19,21,32)	121.07		
			A41	A(19,23,28)	118.77		
			A42	A(1,24,25)	111.20		
			A43	A(1,24,26)	111.39		
			A44	A(1,24,27)	111.35		
			A45	A(25,24,26)	107.17		
			A46	A(25,24,27)	107.70		
			A47	A(26,24,27)	107.84		
			A48	A(23,28,29)	111.47		
			A49	A(23,28,30)	111.47		
			A50	A(23,28,31)	105.80		
			A51	A(29,28,30)	109.48		
			A52	A(29,28,31)	109.27		
			A53	A(30,28,31)	109.27		

Table S3. DFT calculated bond lengths, bond angles, and Mulliken charges for **3**

Bond Length	Definition	Value	Bond Angle	Definition	Value	Atom	Mulliken charge
R1	R(1,2)	1.4021	A1	A(2,1,6)	117.69	1C	0.4751
R2	R(1,6)	1.3970	A2	A(2,1,25)	120.82	2C	0.0134
R3	R(1,25)	1.5096	A3	A(6,1,25)	121.48	3C	0.1413
R4	R(2,3)	1.3881	A4	A(1,2,3)	121.37	4C	-0.4610
R5	R(2,7)	1.0859	A5	A(1,2,7)	119.42	5C	-0.1673
R6	R(3,4)	1.4033	A6	A(3,2,7)	119.21	6C	-0.5976
R7	R(3,8)	1.0838	A7	A(2,3,4)	120.66	7H	0.1758
R8	R(4,5)	1.4019	A8	A(2,3,8)	120.84	8H	0.1591
R9	R(4,11)	1.4052	A9	A(4,3,8)	118.50	9H	0.1591
R10	R(5,6)	1.3936	A10	A(3,4,5)	118.30	10H	0.1380
R11	R(5,9)	1.0842	A11	A(3,4,11)	118.14	11N	0.2095
R12	R(6,10)	1.0856	A12	A(5,4,11)	123.50	12C	-0.0182
R13	R(11,12)	1.2780	A13	A(4,5,6)	120.47	13H	0.0866
R14	R(12,13)	1.0993	A14	A(4,5,9)	119.78	14C	0.6971
R15	R(12,14)	1.4635	A15	A(6,5,9)	119.71	15C	-0.4841
R16	R(14,15)	1.4049	A16	A(1,6,5)	121.47	16C	-0.0866
R17	R(14,16)	1.4008	A17	A(1,6,10)	119.41	17C	-0.4784
R18	R(15,17)	1.3848	A18	A(5,6,10)	119.12	18H	0.1591
R19	R(15,18)	1.0855	A19	A(4,11,12)	120.24	19C	0.0468
R20	R(16,19)	1.3893	A20	A(11,12,13)	121.37	20H	0.1895
R21	R(16,20)	1.0833	A21	A(11,12,14)	123.22	21C	-0.6221
R22	R(17,21)	1.4006	A22	A(13,12,14)	115.40	22H	0.1889
R23	R(17,22)	1.0831	A23	A(12,14,15)	119.74	23H	0.1914
R24	R(19,21)	1.4023	A24	A(12,14,16)	122.00	24O	-0.1522
R25	R(19,23)	1.0817	A25	A(15,14,16)	118.26	25C	-0.5680
R26	R(21,24)	1.3606	A26	A(14,15,17)	121.22	26H	0.1571
R27	R(24,29)	1.4231	A27	A(14,15,18)	119.44	27H	0.1500
R28	R(25,26)	1.0950	A28	A(17,15,18)	119.34	28H	0.1484
R29	R(25,27)	1.0944	A29	A(14,16,19)	121.17	29C	-0.3354
R30	R(25,28)	1.0920	A30	A(14,16,20)	118.60	30H	0.1745
R31	R(29,30)	1.0885	A31	A(19,16,20)	120.23	31H	0.1551
R32	R(29,31)	1.0950	A32	A(15,17,21)	119.86	32H	0.1553
R33	R(29,32)	1.0950	A33	A(15,17,22)	121.47		
			A34	A(21,17,22)	118.67		
			A35	A(16,19,21)	119.76		
			A36	A(16,19,23)	119.35		
			A37	A(21,19,23)	120.90		
			A38	A(17,21,19)	119.73		
			A39	A(17,21,24)	115.84		
			A40	A(19,21,24)	124.44		
			A41	A(21,24,29)	118.89		
			A42	A(1,25,26)	111.24		
			A43	A(1,25,27)	111.40		
			A44	A(1,25,28)	111.33		
			A45	A(26,25,27)	107.16		
			A46	A(26,25,28)	107.69		
			A47	A(27,25,28)	107.81		
			A48	A(24,29,30)	105.75		
			A49	A(24,29,31)	111.35		
			A50	A(24,29,32)	111.35		
			A51	A(30,29,31)	109.37		
			A52	A(30,29,32)	109.36		
			A53	A(31,29,32)	109.57		

Table S4. DFT calculated bond lengths, bond angles, and Mulliken charges for **4**

Name	Definition	Bond Length	Name	Definition	Bond Angle	Atom	Mulliken charge
R1	R(1,2)	1.3981	A1	A(2,1,6)	119.90	1C	-0.3570
R2	R(1,6)	1.3932	A2	A(2,1,7)	120.04	2C	-0.2161
R3	R(1,7)	1.0843	A3	A(6,1,7)	120.06	3C	-0.2532
R4	R(2,3)	1.3884	A4	A(1,2,3)	120.31	4C	1.0133
R5	R(2,8)	1.0843	A5	A(1,2,8)	119.86	5C	-0.5199
R6	R(3,4)	1.4040	A6	A(3,2,8)	119.83	6C	-0.3964
R7	R(3,9)	1.0831	A7	A(2,3,4)	120.22	7H	0.1623
R8	R(4,5)	1.4014	A8	A(2,3,9)	121.14	8H	0.1780
R9	R(4,12)	1.4685	A9	A(4,3,9)	118.63	9H	0.1963
R10	R(5,6)	1.3928	A10	A(3,4,5)	119.06	10H	0.1641
R11	R(5,10)	1.0856	A11	A(3,4,12)	121.68	11H	0.1643
R12	R(6,11)	1.0841	A12	A(5,4,12)	119.25	12C	-0.4044
R13	R(12,13)	1.0989	A13	A(4,5,6)	120.64	13H	0.0925
R14	R(12,14)	1.2764	A14	A(4,5,10)	119.44	14N	0.2200
R15	R(14,15)	1.4058	A15	A(6,5,10)	119.92	15C	-0.3636
R16	R(15,16)	1.4028	A16	A(1,6,5)	119.86	16C	0.1656
R17	R(15,17)	1.4019	A17	A(1,6,11)	120.16	17C	-0.2282
R18	R(16,18)	1.3883	A18	A(5,6,11)	119.98	18C	-0.0568
R19	R(16,19)	1.0838	A19	A(4,12,13)	115.48	19H	0.1646
R20	R(17,20)	1.3931	A20	A(4,12,14)	122.96	20C	-0.4482
R21	R(17,21)	1.0842	A21	A(13,12,14	121.56	21H	0.1561
R22	R(18,22)	1.4018	A22	A(12,14,15	120.36	22C	0.3439
R23	R(18,23)	1.0858	A23	A(14,15,16	118.04	23H	0.1748
R24	R(20,22)	1.3974	A24	A(14,15,17	123.49	24H	0.1478
R25	R(20,24)	1.0856	A25	A(16,15,17	118.41	25C	-0.5547
R26	R(22,25)	1.5095	A26	A(15,16,18	120.60	26H	0.1640
R27	R(25,26)	1.0953	A27	A(15,16,19	118.53	27H	0.1459
R28	R(25,27)	1.0920	A28	A(18,16,19	120.86	28H	0.1451
R29	R(25,28)	1.0938	A29	A(15,17,20	120.41		
			A30	A(15,17,21	119.82		
			A31	A(20,17,21	119.74		
			A32	A(16,18,22	121.35		
			A33	A(16,18,23	119.22		
			A34	A(22,18,23	119.43		
			A35	A(17,20,22	121.45		
			A36	A(17,20,24	119.14		
			A37	A(22,20,24	119.41		
			A38	A(18,22,20	117.73		
			A39	A(18,22,25	120.85		
			A40	A(20,22,25	121.40		
			A41	A(22,25,26	111.24		
			A42	A(22,25,27	111.36		
			A43	A(22,25,28	111.33		
			A44	A(26,25,27	107.59		
			A45	A(26,25,28	107.19		
			A46	A(27,25,28	107.93		

Table S5. DFT calculated bond lengths, bond angles, and Mulliken charges for **5**

Name	Definition	Bond Length	Name	Definition	Bond Angle	Atom	Mulliken Charge
R1	R(1,2)	1.3982	A1	A(2,1,6)	119.89	1C	-0.2620
R2	R(1,6)	1.3933	A2	A(2,1,7)	120.05	2C	-0.3125
R3	R(1,7)	1.0843	A3	A(6,1,7)	120.07	3C	0.0016
R4	R(2,3)	1.3881	A4	A(1,2,3)	120.33	4C	0.5004
R5	R(2,8)	1.0843	A5	A(1,2,8)	119.85	5C	-0.4732
R6	R(3,4)	1.4045	A6	A(3,2,8)	119.82	6C	-0.4950
R7	R(3,9)	1.0829	A7	A(2,3,4)	120.24	7H	0.1513
R8	R(4,5)	1.402	A8	A(2,3,9)	121.19	8H	0.1843
R9	R(4,12)	1.4675	A9	A(4,3,9)	118.57	9H	0.1809
R10	R(5,6)	1.3926	A10	A(3,4,5)	119.00	10H	0.1564
R11	R(5,10)	1.0856	A11	A(3,4,12)	121.77	11H	0.1709
R12	R(6,11)	1.0841	A12	A(5,4,12)	119.23	12C	-0.0799
R13	R(12,13)	1.0977	A13	A(4,5,6)	120.68	13H	0.0671
R14	R(12,14)	1.2772	A14	A(4,5,10)	119.44	14N	0.1559
R15	R(14,15)	1.4103	A15	A(6,5,10)	119.88	15C	0.0984
R16	R(15,16)	1.4063	A16	A(1,6,5)	119.86	16C	0.2321
R17	R(15,17)	1.4024	A17	A(1,6,11)	120.15	17C	-0.1923
R18	R(16,18)	1.3902	A18	A(5,6,11)	119.98	18C	-0.3200
R19	R(16,19)	1.0832	A19	A(4,12,13)	114.58	19H	0.1031
R20	R(17,20)	1.3916	A20	A(4,12,14)	122.46	20C	-0.2430
R21	R(17,21)	1.0835	A21	A(13,12,14)	122.96	21H	0.1750
R22	R(18,22)	1.3963	A22	A(12,14,15)	122.64	22C	-0.3122
R23	R(18,23)	1.0846	A23	A(14,15,16)	125.94	23H	0.1869
R24	R(20,22)	1.3934	A24	A(14,15,17)	115.63	24H	0.1773
R25	R(20,24)	1.0843	A25	A(16,15,17)	118.43	25H	0.1485
R26	R(22,25)	1.084	A26	A(15,16,18)	120.40		
			A27	A(15,16,19)	120.77		
			A28	A(18,16,19)	118.83		
			A29	A(15,17,20)	121.02		
			A30	A(15,17,21)	117.85		
			A31	A(20,17,21)	121.13		
			A32	A(16,18,22)	120.61		
			A33	A(16,18,23)	119.50		
			A34	A(22,18,23)	119.89		
			A35	A(17,20,22)	120.09		
			A36	A(17,20,24)	119.76		
			A37	A(22,20,24)	120.15		
			A38	A(18,22,20)	119.45		
			A39	A(18,22,25)	120.16		
			A40	A(20,22,25)	120.39		

Table S6. DFT calculated dihedral angles for **1–3**

	**1**			**2**			**3**	
Dihedral Angle	Definition	Value	Dihedral angle	Definition	Value	Dihedral Angle	Definition	Value
D1	D(6,1,2,3)	-0.3	D1	D(6,1,2,3)	0.0	D1	D(6,1,2,3)	-0.1
D2	D(6,1,2,7)	-179.6	D2	D(6,1,2,7)	179.1	D2	D(6,1,2,7)	179.2
D3	D(23,1,2,3)	178.0	D3	D(24,1,2,3)	-179.5	D3	D(25,1,2,3)	-179.5
D4	D(23,1,2,7)	-1.3	D4	D(24,1,2,7)	-0.3	D4	D(25,1,2,7)	-0.2
D5	D(2,1,6,5)	-0.7	D5	D(2,1,6,5)	1.0	D5	D(2,1,6,5)	0.9
D6	D(2,1,6,10)	178.7	D6	D(2,1,6,10)	-178.3	D6	D(2,1,6,10)	-178.4
D7	D(23,1,6,5)	-179.0	D7	D(24,1,6,5)	-179.5	D7	D(25,1,6,5)	-179.6
D8	D(23,1,6,10)	0.4	D8	D(24,1,6,10)	1.2	D8	D(25,1,6,10)	1.1
D9	D(2,1,23,24)	-74.3	D9	D(2,1,24,25)	-65.2	D9	D(2,1,25,26)	-64.3
D10	D(2,1,23,25)	45.2	D10	D(2,1,24,26)	54.2	D10	D(2,1,25,27)	55.2
D11	D(2,1,23,26)	165.8	D11	D(2,1,24,27)	174.7	D11	D(2,1,25,28)	175.5
D12	D(6,1,23,24)	104.0	D12	D(6,1,24,25)	115.3	D12	D(6,1,25,26)	116.3
D13	D(6,1,23,25)	-136.5	D13	D(6,1,24,26)	-125.2	D13	D(6,1,25,27)	-124.2
D14	D(6,1,23,26)	-16.0	D14	D(6,1,24,27)	-4.8	D14	D(6,1,25,28)	-3.9
D15	D(1,2,3,4)	1.8	D15	D(1,2,3,4)	-1.8	D15	D(1,2,3,4)	-1.6
D16	D(1,2,3,8)	-179.1	D16	D(1,2,3,8)	178.9	D16	D(1,2,3,8)	179.0
D17	D(7,2,3,4)	-178.8	D17	D(7,2,3,4)	179.1	D17	D(7,2,3,4)	179.1
D18	D(7,2,3,8)	0.2	D18	D(7,2,3,8)	-0.2	D18	D(7,2,3,8)	-0.2
D19	D(2,3,4,5)	-2.4	D19	D(2,3,4,5)	2.6	D19	D(2,3,4,5)	2.5
D20	D(2,3,4,11)	-179.8	D20	D(2,3,4,11)	179.8	D20	D(2,3,4,11)	179.7
D21	D(8,3,4,5)	178.5	D21	D(8,3,4,5)	-178.1	D21	D(8,3,4,5)	-178.2
D22	D(8,3,4,11)	1.1	D22	D(8,3,4,11)	-0.9	D22	D(8,3,4,11)	-0.9
D23	D(3,4,5,6)	1.5	D23	D(3,4,5,6)	-1.6	D23	D(3,4,5,6)	-1.6
D24	D(3,4,5,9)	-176.4	D24	D(3,4,5,9)	176.1	D24	D(3,4,5,9)	176.2
D25	D(11,4,5,6)	178.7	D25	D(11,4,5,6)	-178.7	D25	D(11,4,5,6)	-178.7
D26	D(11,4,5,9)	0.9	D26	D(11,4,5,9)	-1.0	D26	D(11,4,5,9)	-0.9
D27	D(3,4,11,12)	-141.0	D27	D(3,4,11,12)	143.3	D27	D(3,4,11,12)	143.0
D28	D(5,4,11,12)	41.8	D28	D(5,4,11,12)	-39.6	D28	D(5,4,11,12)	-39.9
D29	D(4,5,6,1)	0.1	D29	D(4,5,6,1)	-0.2	D29	D(4,5,6,1)	-0.1
D30	D(4,5,6,10)	-179.3	D30	D(4,5,6,10)	179.1	D30	D(4,5,6,10)	179.2
D31	D(9,5,6,1)	177.9	D31	D(9,5,6,1)	-177.9	D31	D(9,5,6,1)	-177.9
D32	D(9,5,6,10)	-1.5	D32	D(9,5,6,10)	1.4	D32	D(9,5,6,10)	1.4
D33	D(4,11,12,13)	5.0	D33	D(4,11,12,13)	-3.8	D33	D(4,11,12,13)	-4.0
D34	D(4,11,12,14)	-176.6	D34	D(4,11,12,14)	177.2	D34	D(4,11,12,14)	177.0
D35	D(11,12,14,15)	-165.9	D35	D(11,12,14,15)	178.6	D35	D(11,12,14,15)	178.2
D36	D(11,12,14,16)	16.4	D36	D(11,12,14,16)	-1.5	D36	D(11,12,14,16)	-1.8
D37	D(13,12,14,15)	12.5	D37	D(13,12,14,15)	-0.5	D37	D(13,12,14,15)	-0.9
D38	D(13,12,14,16)	-165.1	D38	D(13,12,14,16)	179.5	D38	D(13,12,14,16)	179.1
D39	D(12,14,15,17)	-178.9	D39	D(12,14,15,17)	-180.0	D39	D(12,14,15,17)	-180.0
D40	D(12,14,15,18)	1.1	D40	D(12,14,15,18)	0.0	D40	D(12,14,15,18)	0.0
D41	D(16,14,15,17)	-1.1	D41	D(16,14,15,17)	0.0	D41	D(16,14,15,17)	0.0
D42	D(16,14,15,18)	178.9	D42	D(16,14,15,18)	180.0	D42	D(16,14,15,18)	180.0
D43	D(12,14,16,19)	177.8	D43	D(12,14,16,19)	179.9	D43	D(12,14,16,19)	179.9
D44	D(12,14,16,28)	2.6	D44	D(12,14,16,20)	0.0	D44	D(12,14,16,20)	-0.1
D45	D(15,14,16,19)	0.2	D45	D(15,14,16,19)	-0.1	D45	D(15,14,16,19)	-0.1

D46	D(15,14,16,28)	-175.0	D46	D(15,14,16,20)	180.0	D46	D(15,14,16,20)	180.0
D47	D(14,15,17,20)	1.1	D47	D(14,15,17,21)	0.0	D47	D(14,15,17,21)	0.0
D48	D(14,15,17,21)	-179.6	D48	D(14,15,17,22)	-180.0	D48	D(14,15,17,22)	-180.0
D49	D(18,15,17,20)	-178.9	D49	D(18,15,17,21)	-180.0	D49	D(18,15,17,21)	-180.0
D50	D(18,15,17,21)	0.4	D50	D(18,15,17,22)	0.0	D50	D(18,15,17,22)	0.0
D51	D(14,16,19,20)	0.7	D51	D(14,16,19,21)	0.1	D51	D(14,16,19,21)	0.1
D52	D(14,16,19,22)	-178.3	D52	D(14,16,19,23)	-180.0	D52	D(14,16,19,23)	-180.0
D53	D(28,16,19,20)	176.1	D53	D(20,16,19,21)	-179.9	D53	D(20,16,19,21)	-180.0
D54	D(28,16,19,22)	-3.0	D54	D(20,16,19,23)	0.0	D54	D(20,16,19,23)	0.0
D55	D(14,16,28,29)	-88.9	D55	D(15,17,21,19)	0.0	D55	D(15,17,21,19)	0.0
D56	D(19,16,28,29)	95.9	D56	D(15,17,21,32)	-180.0	D56	D(15,17,21,24)	-180.0
D57	D(15,17,20,19)	-0.2	D57	D(22,17,21,19)	-180.0	D57	D(22,17,21,19)	-180.0
D58	D(15,17,20,27)	179.1	D58	D(22,17,21,32)	0.0	D58	D(22,17,21,24)	0.0
D59	D(21,17,20,19)	-179.5	D59	D(16,19,21,17)	-0.1	D59	D(16,19,21,17)	0.0
D60	D(21,17,20,27)	-0.2	D60	D(16,19,21,32)	179.9	D60	D(16,19,21,24)	179.9
D61	D(16,19,20,17)	-0.7	D61	D(23,19,21,17)	180.0	D61	D(23,19,21,17)	-180.0
D62	D(16,19,20,27)	179.9	D62	D(23,19,21,32)	0.0	D62	D(23,19,21,24)	0.0
D63	D(22,19,20,17)	178.3	D63	D(16,19,23,28)	-179.9	D63	D(17,21,24,29)	180.0
D64	D(22,19,20,27)	-1.0	D64	D(21,19,23,28)	0.0	D64	D(19,21,24,29)	0.0
D65	D(16,28,29,30)	-59.4	D65	D(19,23,28,29)	-61.3	D65	D(21,24,29,30)	180.0
D66	D(16,28,29,31)	63.5	D66	D(19,23,28,30)	61.3	D66	D(21,24,29,31)	-61.3
D67	D(16,28,29,32)	-178.0	D67	D(19,23,28,31)	-180.0	D67	D(21,24,29,32)	61.3

Table S7. DFT-calculated dihedral angles for **4** and **5**

**4**			**5**		
Name	Definition	Dihedral Angle	Name	Definition	Dihedral Angle
D1	D(6,1,2,3)	-0.1	D1	D(6,1,2,3)	0.0
D2	D(6,1,2,8)	-180.0	D2	D(6,1,2,8)	180.0
D3	D(7,1,2,3)	179.9	D3	D(7,1,2,3)	-180.0
D4	D(7,1,2,8)	0.0	D4	D(7,1,2,8)	0.0
D5	D(2,1,6,5)	0.0	D5	D(2,1,6,5)	0.0
D6	D(2,1,6,11)	-180.0	D6	D(2,1,6,11)	-180.0
D7	D(7,1,6,5)	-180.0	D7	D(7,1,6,5)	180.0
D8	D(7,1,6,11)	0.0	D8	D(7,1,6,11)	0.0
D9	D(1,2,3,4)	0.1	D9	D(1,2,3,4)	0.0
D10	D(1,2,3,9)	-179.9	D10	D(1,2,3,9)	-180.0
D11	D(8,2,3,4)	-180.0	D11	D(8,2,3,4)	-180.0
D12	D(8,2,3,9)	0.0	D12	D(8,2,3,9)	0.0
D13	D(2,3,4,5)	-0.1	D13	D(2,3,4,5)	0.0
D14	D(2,3,4,12)	179.9	D14	D(2,3,4,12)	-180.0
D15	D(9,3,4,5)	179.9	D15	D(9,3,4,5)	-180.0
D16	D(9,3,4,12)	0.0	D16	D(9,3,4,12)	0.0
D17	D(3,4,5,6)	0.0	D17	D(3,4,5,6)	0.0
D18	D(3,4,5,10)	-180.0	D18	D(3,4,5,10)	180.0
D19	D(12,4,5,6)	-180.0	D19	D(12,4,5,6)	180.0
D20	D(12,4,5,10)	0.0	D20	D(12,4,5,10)	0.0
D21	D(3,4,12,13)	179.3	D21	D(3,4,12,13)	-180.0
D22	D(3,4,12,14)	-1.7	D22	D(3,4,12,14)	0.0
D23	D(5,4,12,13)	-0.7	D23	D(5,4,12,13)	0.0
D24	D(5,4,12,14)	178.4	D24	D(5,4,12,14)	-180.0
D25	D(4,5,6,1)	0.0	D25	D(4,5,6,1)	0.0
D26	D(4,5,6,11)	-180.0	D26	D(4,5,6,11)	-180.0
D27	D(10,5,6,1)	-180.0	D27	D(10,5,6,1)	-180.0
D28	D(10,5,6,11)	0.0	D28	D(10,5,6,11)	0.0
D29	D(4,12,14,15)	177.1	D29	D(4,12,14,15)	-180.0
D30	D(13,12,14,15)	-3.8	D30	D(13,12,14,15)	0.0
D31	D(12,14,15,16)	143.2	D31	D(12,14,15,16)	0.0
D32	D(12,14,15,17)	-39.5	D32	D(12,14,15,17)	-180.0
D33	D(14,15,16,18)	179.9	D33	D(14,15,16,18)	180.0
D34	D(14,15,16,19)	-1.0	D34	D(14,15,16,19)	0.0
D35	D(17,15,16,18)	2.5	D35	D(17,15,16,18)	0.0
D36	D(17,15,16,19)	-178.4	D36	D(17,15,16,19)	-180.0
D37	D(14,15,17,20)	-178.8	D37	D(14,15,17,20)	180.0
D38	D(14,15,17,21)	-0.9	D38	D(14,15,17,21)	0.0
D39	D(16,15,17,20)	-1.5	D39	D(16,15,17,20)	0.0
D40	D(16,15,17,21)	176.4	D40	D(16,15,17,21)	180.0
D41	D(15,16,18,22)	-1.8	D41	D(15,16,18,22)	0.0
D42	D(15,16,18,23)	178.9	D42	D(15,16,18,23)	-180.0
D43	D(19,16,18,22)	179.1	D43	D(19,16,18,22)	180.0
D44	D(19,16,18,23)	-0.2	D44	D(19,16,18,23)	0.0
D45	D(15,17,20,22)	-0.1	D45	D(15,17,20,22)	0.0

D46	D(15,17,20,24)	179.3	D46	D(15,17,20,24)	-180.0
D47	D(21,17,20,22)	-178.0	D47	D(21,17,20,22)	-180.0
D48	D(21,17,20,24)	1.4	D48	D(21,17,20,24)	0.0
D49	D(16,18,22,20)	0.2	D49	D(16,18,22,20)	0.0
D50	D(16,18,22,25)	-178.3	D50	D(16,18,22,25)	-180.0
D51	D(23,18,22,20)	179.5	D51	D(23,18,22,20)	180.0
D52	D(23,18,22,25)	1.0	D52	D(23,18,22,25)	0.0
D53	D(17,20,22,18)	0.7	D53	D(17,20,22,18)	0.0
D54	D(17,20,22,25)	179.2	D54	D(17,20,22,25)	180.0
D55	D(24,20,22,18)	-178.7	D55	D(24,20,22,18)	-180.0
D56	D(24,20,22,25)	-0.2	D56	D(24,20,22,25)	0.0
D57	D(18,22,25,26)	70.7			
D58	D(18,22,25,27)	-169.3			
D59	D(18,22,25,28)	-48.8			
D60	D(20,22,25,26)	-107.8			
D61	D(20,22,25,27)	12.2			
D62	D(20,22,25,28)	132.7			

Table S8. Characteristic FT-IR absorption bands (cm^{-1}) of Schiff bases **1**–**5***

ν	**1**		**2**		**3**		**4**		**5**	
(cm^{-1})	Exp	DFT	Exp	DFT	Exp	DFT	Exp	DFT	Exp	DFT
C=N	1623	1647.4	1627	1653.1	1659	1647.4	1627	1647.4	1627	1653.1
C–H (C=N)	3021	3029.8	3001	3030.7	3000	3029.8	3026	3030.0	3029	3029.3
O–CH_3	1255	1248.0	1271	1249.9	1246	1248.0	1242	1248.0	1246	1249.9
O–$C_{Ar'}$	1042	1021.7	1043	1021.4	1022	1017.6	1021	1017.6	1025	1021.4
C–H (Ar')	1598	1601.3	1586	1599.4	1598	1601.3	1602	1601.3	1592	1599.4
C–H (Ar)	1598	1593.6	1586	1592.6	1598	1593.6	1602	1593.6	1592	1592.6
C=C	1502	1501.4	1483	1498.6	1505	1501.4	1505	1501.4	1490	1498.6
C_{Ar}–N	1269	1277.5	1271	1276.8	1303	1276.1	1278	1278.4	1286	1276.8

* Experimental: KBr pellet; DFT: scaled (0.96).

Table S9. Experimental (E) and DFT-computed (T) ^{1}H NMR chemical shifts for **1–5**

^{1}H NMR (δ ppm)	**1**		**2**		**3**		**4**		**5**	
	E	T	E	T	E[a-c]	T	E[a-c]	T	E[b-e]	T
C***H***$_3$	2.4	2.1	2.4	2.1	2.4	2.0	2.4	2.0	-	-
OC**H**$_3$		2.5		2.6		2.5		2.4		
Ar***H***		2.6		2.6		2.5		2.6		
(benzylidene	3.9	3.6	3.9	3.7	3.9	3.8	-	-	-	-
ring)		4.0		3.8		3.8				
		4.6		4.1		4.1				
	7.0	7.5	7.2	7.2	7.0	7.2	7.2	7.6	7.9	7.6
Ar***H***	7.0	7.5	7.4	7.4	7.8	7.2	7.5	7.6		7.6
(aniline ring)	7.4	7.7	7.4	7.7		7.7	7.9	7.6	7.4	7.7
	8.1	7.9	7.4	8.2		8.7		7.7		7.7
								8.8		8.9
C***H***=N	7.2	7.2	7.1	7.2	7.1	7.2	7.1	7.1	7.4	7.4

(*a*) Jiang, L.; Jin, L.; Tian, H.; Yuan, X.; Yu, X.; Xu, Q. Direct and mild palladium-catalyzed aerobic oxidative synthesis of imines from alcohols and amines under ambient conditions. *Chem. Commun.* **2011**, *47*, 10833–10835.

(*b*) Jothi, A. I.; Rajarathinam, C.; Viveke, A. A.; Paul, M. W. B. Substituent effects on the mesogenic benzylidenes of 4-methylaniline: synthesis, characterization, DFT, NLO, photophysical, molecular docking, and antibacterial studies. *J. Mol. Liq.* **2021**, 347, 117980.

(*c*) Han, B.; Jiao, H.; Chen, R.; Zhang, Y.; Wang, J. Chemoselective reduction of imines and azobenzenes catalyzed by silver *N*-heterocyclic carbene complexes. *Org. Chem. Front.* **2023**, *10*, 2287-2293.

(*d*) Yan, D.; Wu, X.; Xiao, J.; Zhu, Z.; Xu, X.; Bao, X.; Yao, Y.; Shen, Q.; Xue, M. *n*-Butyllithium catalysed hydroboration of imines and alkynes. *Org. Chem. Front.* **2019**, *6*, 648-653.

(*e*) Zhang, E.; Tian, H.; Xu, S.; Yu, X.; Xu, Q. Iron-catalysed direct synthesis of imines from amines or alcohols and amines via aerobic oxidative reactions under air. *Org. Lett.* **2013**, *15*, 2704-2707.

Table S10. Experimental (E) and DFT-calculated (T) ^{13}C NMR chemical shifts for **1–5**

^{13}C NMR (δ ppm)	**1**		**2**		**3**		**4**		**5**	
	Exp	DFT	Exp	DFT	Exp[a-c]	DFT	Exp[a-c]	DFT	Exp[b-e]	DFT
CH_3	21	22	21	22	21	21	21	22	-	-
O***C***H_3	56	64	55	56	55	56	-	-	-	-
C$_{Ar}$-CH_3	136	142	136	143	135	142	136	141	-	-
C$_{Ar'}$-OCH_3	159	168	160	167	162	170	-	-	-	-
C$_{Ar}$-C=N	125	136	138	144	129	135	136	143	136	143
C$_{Ar}$-N=C	150	158	149	157	150	157	149	157	152	154
CH=N	156	165	160	165	159	164	160	163	160	158
C$_{Ar'}$H	111	128	112	116	115	113	129	131	121	131
	121	130	118	118	130	121	129	132	126	132
	128	137	122	134		133	132	133	129	132
	133	142	130	144		139		137		133
										137
C$_{Ar}$H	121	120	122	120	121	120	121	119	129	116
	130	130	130	130	130	130	130	130	129	132
		134		134		134		134	131	132
		134		134		134		134		134
										138

(*a*) Jiang, L.; Jin, L.; Tian, H.; Yuan, X.; Yu, X.; Xu, Q. Direct and mild palladium-catalyzed aerobic oxidative synthesis of imines from alcohols and amines under ambient conditions. *Chem. Commun.* **2011**, *47*, 10833–10835.

(*b*) Jothi, A. I.; Rajarathinam, C.; Viveke, A. A.; Paul, M. W. B. Substituent effects on the mesogenic benzylidenes of 4-methylaniline: synthesis, characterization, DFT, NLO, photophysical, molecular docking, and antibacterial studies. *J. Mol. Liq.* **2021**, 347, 117980.

(*c*) Han, B.; Jiao, H.; Chen, R.; Zhang, Y.; Wang, J. Chemoselective reduction of imines and azobenzenes catalyzed by silver *N*-heterocyclic carbene complexes. *Org. Chem. Front.* **2023**, *10*, 2287-2293.

(*d*) Yan, D.; Wu, X.; Xiao, J.; Zhu, Z.; Xu, X.; Bao, X.; Yao, Y.; Shen, Q.; Xue, M. *n*-Butyllithium catalysed hydroboration of imines and alkynes. *Org. Chem. Front.* **2019**, *6*, 648-653.

(*e*) Zhang, E.; Tian, H.; Xu, S.; Yu, X.; Xu, Q. Iron-catalysed direct synthesis of imines from amines or alcohols and amines via aerobic oxidative reactions under air. *Org. Lett.* **2013**, *15*, 2704-2707.

Table S11. Electronic absorption spectral data for **1–5** in different solvents

Solvent	$E_T(30)^a$	Absorption band	**1**		**2**		**3**		**4**		**5**	
			λ (nm)	A	λ (nm)	A	λ (nm)	A	λ (nm)	A	λ (nm)	A
n-Hexane	30.9	π-π*	242.8	0.462	242.8	0.785	279.9	0.844	268.7	0.056	261.3	0.332
		π-π*					287.3	0.664				
		n-π*	305.9	0.180	289.8	0.142	320.8	0.397			309.6	0.128
Dichloromethane	40.7	π-π*	266.3	0.087	240.3	0.217	241.6	0.498				
		n-π*	331.9	0.088	299.7	0.079	282.4	0.800				
Chloroform	39.1	π-π*									263.8	1.112
		n-π*	322.0	0.927	296.0	0.086	282.4	0.800	283.6	0.245	310.9	0.505
Ethyl acetate	38.1	π-π*					268.8	0.632				
		n-π*	309.6	0.731	298.5	0.166						
Methanol	55.4	π-π*	261.3	0.940	241.5	0.852	282.4	0.812				
		n-π*	328.2	0.95	296.0	0.13	319.5	0.769				
Ethanol	51.9	π-π*	265.0	0.285	236.6	0.540	282.4	0.704	241.6	1.01	245.2	0.598
		n-π*	330.7	0.291	294.6	0.108	320.8	0.729	282.4	0.131	283.6	0.103
Acetonitrile	45.6	π-π*			248.9	0.754			240.3	0.634		
		π-π*	266.2	0.263			272.4	0.707	266.2	0.474	262.3	1.131
		n-π*	330.7	0.247	283.6	0.150	369.1	0.253	322.0	0.276	310.5	0.513
DMSO	45.1	π-π*			296.0	0.272						
		n-π*	333.3	0.575	386.3	0.007						
Water	63.1	π-π*	253.9	0.698	230.4	0.145	223.0	0.530				
		n-π*	320.8	0.252	293.5	0.044	284.8	0.525				

(*a*) Reichardt, C. Solvatochromic dyes as solvent polarity indicators. *Chem. Rev.* **1994**, *94*, 2319-2358.

Table S12. TD-DFT simulated UV–Vis absorption spectral data for **1**–**5** in different solvents

Solvent medium →		Gas phase		Chloroform		Ethanol		Acetonitrile	
Schiff base	Absorption band	*λ* (nm)	*ε* *(a.u.)*	*λ* (nm)	*ε* *(a.u.)*	*λ* (nm)	*ε* *(a.u.)*	*λ* (nm)	*ε* *(a.u.)*
**1**	*π-π**	274.4	14248			284.5	20425	284.5	20580
	*n-π**	347.2	12649	353.8	20999	356.5	24285	356.5	24542
**2**	*π-π**	275.2	17386	282.7	18785	287.2	11363	280.9	18193
	*n-π**	344.8	15100	352.0	24641	354.7	28384	354.7	28719
**3**	*π-π**	292.8	18342						
	*n-π**	338.2	21352	350.4	34099	354.7	38322	354.7	38653
**4**	*π-π**	275.2	17242	278.4	18574			277.6	17944
	*n-π**	345.6	14121	348.0	20085	336.0	28306	345.6	19943
**5**	*π-π**	293.3	8572					263.2	15425
	*n-π**	317.7	32915	348.0	31763	350.0	36893	441.0	46957

Table S13. DFT calculated Mulliken atomic charges for key atoms of Schiff bases **1**–**5**

Atom	**1**	**2**	**3**	**4**	**5**
**H**C=N	0.082	0.086	0.087	0.092	0.067
**C**=N	0.502	-0.403	-0.018	-0.404	-0.08
C=**N**	0.164	0.242	0.209	0.22	0.156
$\mathbf{C}_{Ar'}$	1.026	1.256	0.697	1.013	0.500
$\mathbf{C}_{Ar}$	-0.707	-0.301	-0.461	-0.364	-0.098
$\mathbf{C}_{Ar}$Me	0.412	0.312	0.475	0.344	
$\mathbf{C}H_3$	-0.498	-0.57	-0.568	-0.555	
$\mathbf{C}_{Ar'}$-OMe	-1.068	-0.346	-0.622		
**O**Me	-0.08	-0.151	-0.152		
$O\mathbf{C}H_3$	-0.284	-0.308	-0.335		

Table S14. DFT-calculated frontier orbital energies and global reactivity descriptors of **1**–**5**

**Electronic parameters***	**1**	**2**	**3**	**4**	**5**
HOMO (eV)	4.05	6.02	5.82	6.07	6.26
LUMO (eV)	2.35	1.84	1.68	1.90	2.08
ΔE (eV)	1.70	4.18	4.15	4.17	4.18
Ionization potential (I, eV)	4.05	6.02	5.82	6.07	6.26
Electron affinity (A, eV)	2.35	1.84	1.68	1.90	2.08
Chemical potential (μ, eV)	3.20	3.93	3.75	3.98	4.17
Absolute electronegativity (χ, eV)	2.77	2.88	2.71	2.94	3.13
Absolute hardness (η, eV)	0.85	2.09	2.07	2.08	2.09
Absolute softness (σ, eV^{-1})	1.17	0.48	0.48	0.48	0.48

* $I = E_{HOMO}$, $A = E_{LUMO}$, μ = - (I+A)/2), χ = (I+A)/2), η = (I-A)/2, $\sigma = 1/\eta$.

Table S15. DFT-calculated dipole moment (μ), linear polarizability (α), and first hyperpolarizability (β) of **1**–**5**

Schiff base	Dipole moment (D)	Polarizability (x 10^{-24} esu)	Hyperpolarizability (x 10^{-30} esu)
**1**	1.6	31.3	2.4
**2**	2.1	32.0	3.0
**3**	1.5	32.7	6.7
**4**	1.3	29.0	2.1
**5**	1.3	29.3	1.8
